# A semi-symmetric image encryption scheme based on the function projective synchronization of two hyperchaotic systems

**DOI:** 10.1371/journal.pone.0184586

**Published:** 2017-09-14

**Authors:** Xiaoqiang Di, Jinqing Li, Hui Qi, Ligang Cong, Huamin Yang

**Affiliations:** School of Computer Science and Technology, Changchun University of Science and Technology, Changchun, JiLin Province, China; Kaohsiung Medical University, TAIWAN

## Abstract

Both symmetric and asymmetric color image encryption have advantages and disadvantages. In order to combine their advantages and try to overcome their disadvantages, chaos synchronization is used to avoid the key transmission for the proposed semi-symmetric image encryption scheme. Our scheme is a hybrid chaotic encryption algorithm, and it consists of a scrambling stage and a diffusion stage. The control law and the update rule of function projective synchronization between the 3-cell quantum cellular neural networks (QCNN) response system and the 6^*th*^-order cellular neural network (CNN) drive system are formulated. Since the function projective synchronization is used to synchronize the response system and drive system, Alice and Bob got the key by two different chaotic systems independently and avoid the key transmission by some extra security links, which prevents security key leakage during the transmission. Both numerical simulations and security analyses such as information entropy analysis, differential attack are conducted to verify the feasibility, security, and efficiency of the proposed scheme.

## Introduction

With the rapid growth of broadband communication, multimedia transmission has increased over the Internet, which makes information and communication systems more vulnerable. Image security has attracted a huge amount of attention due to the widespread interconnection of almost all devices and communication networks. Image encryption differs from text encryption due to bulk data capacity, high redundancy and a strong correlation between adjacent pixels.

Since Matthews [1] first proposed the chaos encryption algorithm in 1989, many studies have indicated that chaotic encryption are suitable for bulk data due to its favorable properties, such as complex and nonlinear, high sensitivity to initial conditions, control parameters, non-periodicity, and a pseudorandom nature.

Many image encryption algorithms [2–25] based on chaos have been developed in last decades to ensure the security of digital images transmission and storage. Most of them adopted permutation-diffusion mechanism [3–7, 11, 16, 17, 19, 21], in which permuting the positions of image pixels incorporates with changing gray values of image pixels to confuse the relationship between the cipher image and the plain image.

A previous study [26]proposed an image encryption/decryption algorithm with compound chaos mapping, in addition, a hyperchaotic system based on chaotic control parameters was put forward. Another study [27] presented an image encryption scheme on the foundation of multiple chaotic maps while an alternate work [28] proposed an image encryption algorithm on the basis of rotation matrix bit-level permutation and block diffusion. Akram Belazi proposed several image encryption schemes [23–25] based on chaos and obtained the good encryption effect. An encryption method on the basis of reversible cellular automata combined with chaos has also been designed in [12]. Ref [29] presented a color image encryption scheme on the foundation of the quantum chaotic system.

According to the type of the key usage, encryption algorithm can be divided into symmetric encryption and asymmetric encryption. The same secret key is used to encrypt and decrypt in symmetric encryption algorithms. Most chaos image encryption schemes are based on symmetric cryptographic techniques, which have been proven to be more vulnerable than an asymmetric cryptosystem [30].

Common symmetric encryption algorithms include DES, 3DES and AES. They are widely used due to their advantages such as great speed, relatively low complexity as well as easy implementation in hardware. Since both encryption and decryption sides should configure the key by some extra methods, once the key is divulged the cryptosystems will be broken. Furthermore, each pair of users need choose a unique key that nobody else knows. This makes the quantity of key to be growing exponentially.

Asymmetric encryption differs from symmetric encryption that it requires a key pair: a public key for encryption and a corresponding private key for decryption which is known only to the owner. The most common asymmetric encryption algorithm is RSA. In an asymmetric key cryptosystem, any user can encrypt a message using the public key of the receiver, but such a message can be decrypted only with the receiver’s private key [31]. It is unlike symmetric encryption to share the key, asymmetric encryption do not require a secure channel for the initial exchange of the key from transmitter to receiver. Although asymmetric cryptosystem has so many advantages, it also has disadvantages. For example, it is extremely difficult to factorize large numbers in order to obtain sufficiently long keys especially enormous data.

Since Pecora and Corrall found the drive-response chaos synchronization phenomena [32], a lot of synchronization schemes have been proposed, such as complete synchronization, generalized synchronization, phase synchronization, lag synchronization, projective synchronization. Two chaotic systems synchronization phenomenon is similar to the asymmetric key mechanism, and they can synchronize with each other if they exchange information in just the right way. This motivates us to use chaos synchronization to avoid the key transmission in order to combine the advantage of the symmetric and asymmetric encryption and try to overcome their shortcomings.

In this paper, we propose a new color image cryptosystem using a synchronization scheme for a 3-cell QCNN [33] and a 6^*th*^-order CNN [34]. The 3-cell QCNN is regarded as the response system and the 6th order CNN is used for the drive system. In order to synchronize the drive-response system, the control law for stable synchronization errors and the update rule for unknown parameters estimation are given. The function projective synchronization [35] is treated as the decryption key generator. We prove that the 6^*th*^-order CNN drive system and the 3-cell QCNN response system are asymptotically synchronized. Numerical simulations and security analyses such as information entropy analysis, differential attack are performed to verify the feasibility of the proposed scheme. As similar as the asymmetric encryption, our scheme does not require exchange key, and it effectively avoids the threat of key exposure, therefore, it will be called Semi-Symmetric encryption scheme.

The rest of the paper is organized as follows: In the next section, we briefly describe the 3-cell QCNN system and the 6^*th*^-order CNN system used in our scheme. In Section 3, the function projection synchronization between the response system and the drive system is presented. Section 4 gives the semi-symmetric encryption scheme. The experimental results and performance analyses are given in Section 5. Section 6 concludes the paper.

## System descriptions

### 3-cell QCNN hyperchaotic system

**Fig 1:**
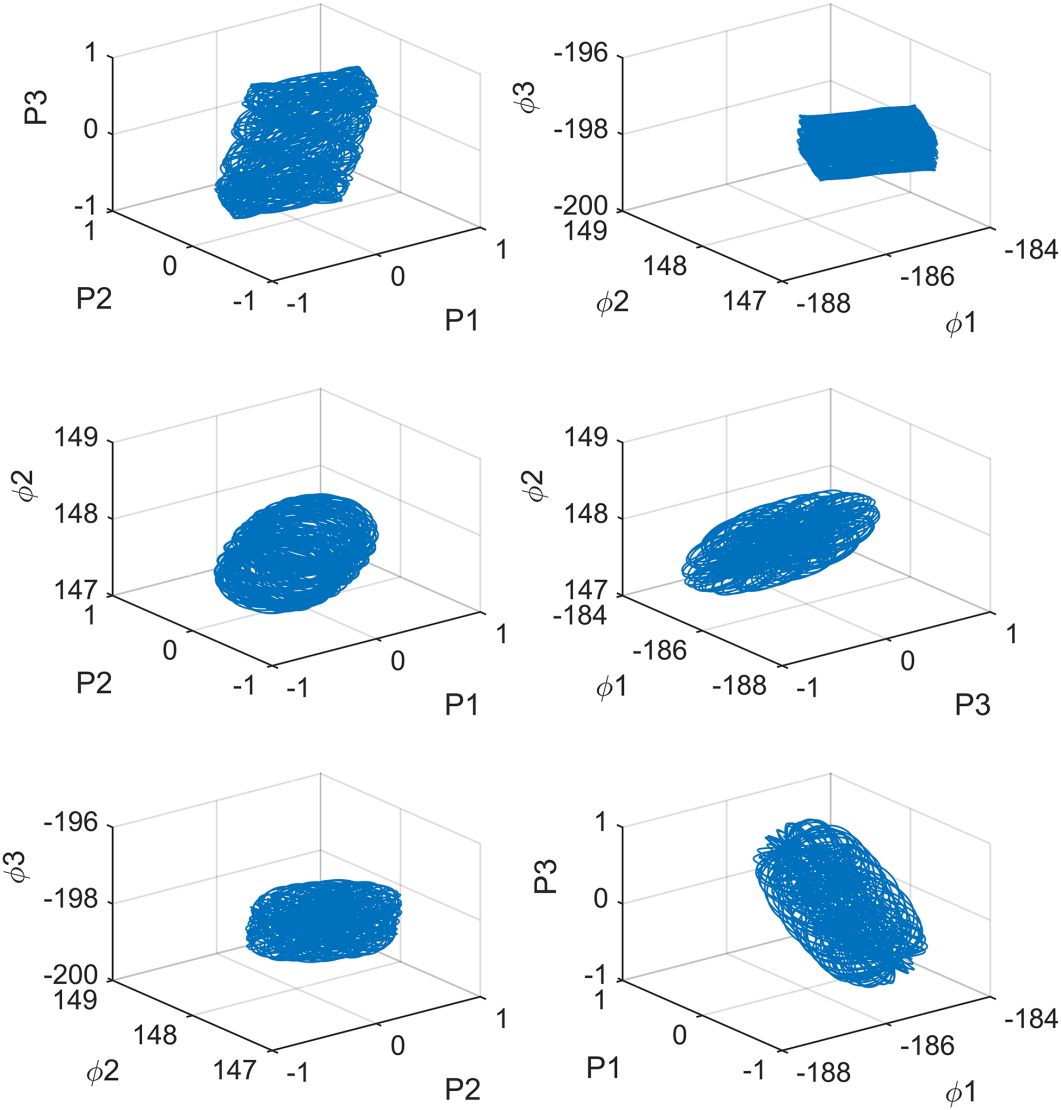
3-cell QCNN system partial attractor distribution.

**Fig 2:**
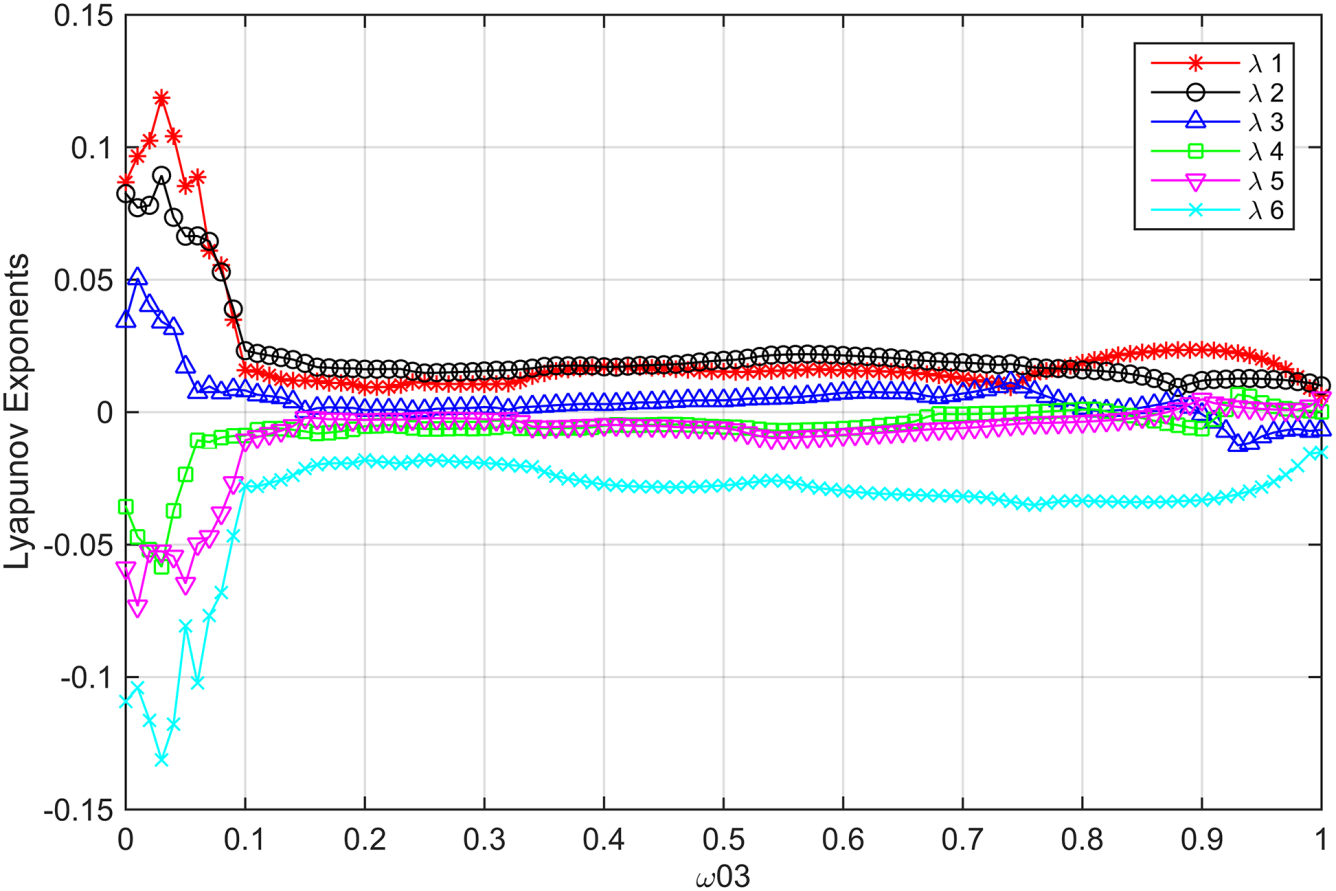
3-cell QCNN system Lyapunov exponents spectrum with *b*_01_ = *b*_02_ = *b*_03_ = 0.28, *ω*_01_ = 0.5, *ω*_02_ = 0.2, and *ω*_03_ ∈ [0, 1].

Quantum dots and quantum cellular automata (QCA) [36] constitute new types of semiconductor nano-materials that have many unique nano-features. The k^*th*^ QCA state equation is obtained by the Schrödinger equation [36]:

$$\begin{array}{ccc} i\hbar \frac{\partial }{\partial t}P_{k} & = & -2\gamma \sqrt{1-P_{k}^{2}}\sin\varphi_{k}\\ i\hbar \frac{\partial }{\partial t}\varphi {}_{k} & = & -E_{k}{\bar{P}}_{k}+2\gamma \frac{P_{k}}{\sqrt{1-P_{k}^{2}}}\cos\varphi_{k} \end{array}$$ (1)

where ℏ is Planck’s constant, *γ* is the inter-dot tunneling energy, which takes into account the neighboring polarizations, and *E*_*k*_ is the electrostatic energy cost of two adjacent fully polarized cells that have opposite polarization. The effect of local interconnections is considered in the term ${\bar{P}}_{k}$; and *φ*_*k*_ is a quantum phase of the QCA. Eq (1) constitutes the QCNN state equations and its dynamics are characterized by two variables, *P*_*k*_ and *ϕ*_*k*_. A 3–cell QCNN system can be described as Eq (2):

$$\begin{array}{ccc} {\dot{P}}_{1} & = & -2b_{01}\sqrt{1-P_{1}^{2}}\sin\phi_{1}\\ {\dot{\phi }}_{1} & = & -\omega_{01}\left(P_{1}-P_{2}-P_{3}\right)+2b_{01}\frac{P_{1}}{\sqrt{1-P_{1}^{2}}}\cos\phi_{1}\\ {\dot{P}}_{2} & = & -2b_{02}\sqrt{1-P_{2}^{2}}\sin\phi_{2}\\ {\dot{\phi }}_{2} & = & -\omega_{02}\left(P_{2}-P_{1}-P_{3}\right)+2b_{02}\frac{P_{2}}{\sqrt{1-P_{2}^{2}}}\cos\phi_{2}\\ {\dot{P}}_{3} & = & -2b_{03}\sqrt{1-P_{3}^{2}}\sin\phi_{3}\\ {\dot{\phi }}_{3} & = & -\omega_{03}\left(P_{3}-P_{1}-P_{2}\right)+2b_{03}\frac{P_{3}}{\sqrt{1-P_{3}^{2}}}\cos\phi_{3} \end{array}$$ (2)

where *P*_1_, *P*_2_, *P*_3_ and *ϕ*_1_, *ϕ*_2_, *ϕ*_3_ are the state variables; *b*_01_, *b*_02_, and *b*_03_ are the proportional inter–dot energy in each cell, and *ω*_01_, *ω*_02_, *ω*_03_ are effect weigh parameters on the differences in the polarization of the adjacent cells, like the cloning templates in traditional CNNs. The Fig 1, shows the attractor of system(2) in three dimensional space. We investigated the dynamic behavior of system(2) by calculating its Lyapunov exponents. When *b*_01_ = *b*_02_ = *b*_03_ = 0.28, *ω*_01_ = 0.5, *ω*_02_ = 0.2, and *ω*_03_ ∈ [0, 1], which are shown in Fig 2, system(2) is hyperchaotic due to three positive Lyapunov exponents.

### 6^*th*^-order CNN hyperchaotic system

The 6^*th*^-order CNN is another hyperchaotic system used in this paper, which is introduced in Ref [34], and it is all the interconnection in a CNN. Its state equation is defined as Eq (3):

$$\begin{array}{c} \frac{dx_{i}}{dt}=-x_{j}+a_{j}p_{j}+\sum_{\begin{array}{c} k=1\\ k\neq j \end{array}}^{6}a_{j,k}p_{k}+\sum_{k=1}^{6}s_{jk}x_{k}+i_{j}\;\left(j=1,2,...,6\right) \end{array}$$ (3)

where

$$\begin{array}{c} \begin{array}{c} a_{j}=0\left(j=1,2,3,5,6\right),a_{4}=200;\\ a_{jk}=0\left(j,k=1,2,...,6;j\neq k\right);\\ s_{12}=s_{21}=s_{24}=s_{34}=s_{42}=s_{43}=s_{53}=s_{54}=s_{55}=s_{56}=s_{61}=s_{63}=s_{64}=0;\\ i_{j}=0\left(j=1,2,...,6\right);\\ s_{11}=s_{23}=s_{33}=s_{51}=1;s_{13}=s_{14}=-1;\\ s_{22}=3,s_{31}=14,s_{32}=-14,s_{41}=s_{62}=100,s_{44}=-99,s_{52}=18,s_{65}=4,s_{66}=-3; \end{array} \end{array}$$

Eq (3) could be calculted as Eq (4):

$$\begin{array}{ccc} {\dot{x}}_{1} & = & -x_{3}-x_{4}\\ {\dot{x}}_{2} & = & 2x_{2}+x_{3}\\ {\dot{x}}_{3} & = & 14x_{1}-14x_{2}\\ {\dot{x}}_{4} & = & 100x_{1}-100x_{4}+200p{}_{4}\\ {\dot{x}}_{5} & = & 18x_{2}+x_{1}-x_{5}\\ {\dot{x}}_{6} & = & 4x_{5}-4x_{6}+100x_{2} \end{array}$$ (4)

where *p*_4_ = 0.5(|*x*_4_ + 1| − |*x*_4_ − 1|).

**Fig 3:**
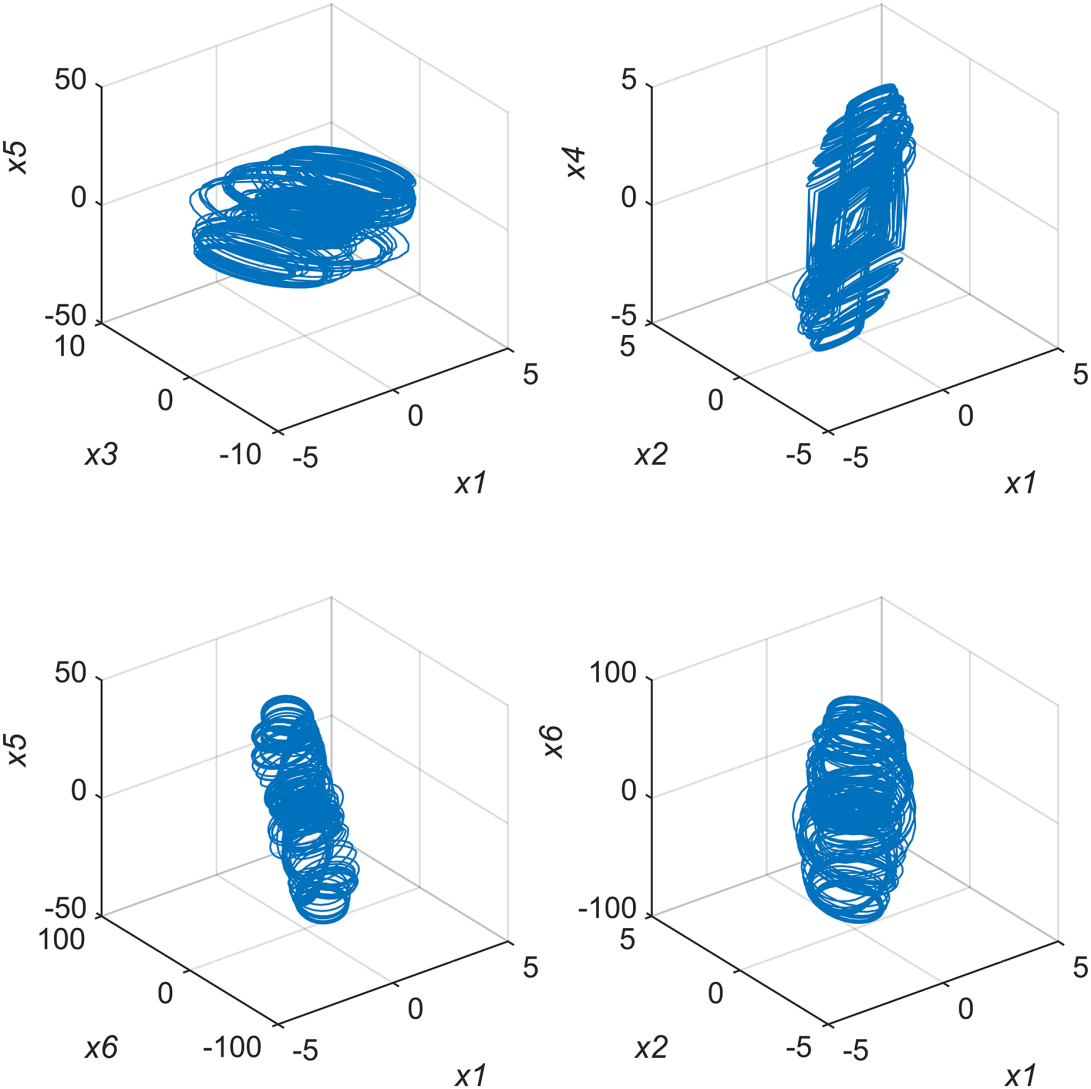
6^*th*^-order CNN partial chaotic attractor distribution.

We calculated the Lyapunov exponents of system(4). When *t* → ∞, the six Lyapunov exponents are *λ*_1_ = 2.748, *λ*_2_ = −2.9844, *λ*_3_ = 1.2411, *λ*_4_ = −14.4549, *λ*_5_ = −1.4123 and *λ*_6_ = −83.2282. Two of these exponents are positive, so system(4) is also hyperchaotic. Fig 3 shows system(4)’s partial chaotic attractor distribution.

### The synchronized key generation system

Let System(4) and System(2) be the drive system and the response system, respectively. Thus, the system(2) can be described by the Eq (5) via the function projective synchronization [35]:

$$\begin{array}{ccc} {\dot{P}}_{r1} & = & -2b_{11}\sqrt{1-P_{r1}^{2}}\sin\phi_{r1}+u_{1}\\ {\dot{\phi }}_{r1} & = & -\omega_{11}\left(P_{r1}-P_{r2}-P_{r3}\right)+2b_{11}\frac{P_{r1}}{\sqrt{1-P_{r1}^{2}}}\cos\phi_{r1}+u_{2}\\ {\dot{P}}_{r2} & = & -2b_{12}\sqrt{1-P_{r2}^{2}}\sin\phi_{r2}+u_{3}\\ {\dot{\phi }}_{r2} & = & -\omega_{12}\left(P_{r2}-P_{r1}-P_{r3}\right)+2b_{12}\frac{P_{r2}}{\sqrt{1-P_{r2}^{2}}}\cos\phi_{r2}+u_{4}\\ {\dot{P}}_{r3} & = & -2b_{13}\sqrt{1-P_{r3}^{2}}\sin\phi_{r3}+u_{5}\\ {\dot{\phi }}_{r3} & = & -\omega_{13}\left(P_{r3}-P_{r1}-P_{r2}\right)+2b_{13}\frac{P_{r3}}{\sqrt{1-P_{r3}^{2}}}\cos\phi_{r3}+u_{6} \end{array}$$ (5)

where *b*_11_, *b*_12_, *b*_13_, *ω*_11_, *ω*_12_ and *ω*_13_ are the parameters of response system(5) that need to be estimated in order to synchronize system(4) and system(5), and *u*_1_, *u*_2_, *u*_3_, *u*_4_, *u*_5_ and *u*_6_ are the controllers. Define synchronization error states as follows:

$$\begin{array}{c} {\dot{e}}_{i}={\dot{y}}_{i}-\alpha \left(t\right){\dot{x}}_{i}-\dot{\alpha }\left(t\right)x_{i},i=1,2,3,4,5,6 \end{array}$$ (6)

which ${\dot{e}}_{i}$ denotes the deviation between system(4) and system(5), when ${\dot{e}}_{i}$ converges to zero as time tends to infinity $\lim_{t\to \infty }\left|\right|e_{i}\left|\right|=\lim_{t\to \infty }\left|\right|y_{i}-\alpha \left(t\right)x_{i}\left|\right|=0,i=1,2,3,4,5,6\;$, *α*(*t*) as the scaling function factor, drive system and response system reach synchronization. Substituting Eqs (2), (4) and (5) into Eq (6) yields the error dynamical system(7) as defined in Eq (7) between system(4) and system(5):

$$\begin{array}{ccc} {\dot{e}}_{1} & = & -2b_{11}\sqrt{1-P_{r1}^{2}}\sin\varphi_{r1}+u_{1}-\alpha \left(t\right)\left(-2b_{01}\sqrt{1-P_{1}^{2}}\sin\varphi_{1}\right)-\dot{\alpha }\left(t\right)P_{1}\\ {\dot{e}}_{2} & = & -\omega_{11}\left(P_{r1}-P_{r2}-P_{r3}\right)+2b_{11}\frac{P_{r1}}{\sqrt{1-P_{r1}^{2}}}\cos\varphi_{r1}+u_{2}\\ & & -\alpha \left(t\right)\left[-\omega_{01}\left(P_{1}-P_{2}-P_{3}\right)+2b_{01}\frac{P_{1}}{\sqrt{1-P_{1}^{2}}}\cos\varphi_{1}\right]-\dot{\alpha }\left(t\right)\varphi_{1}\\ {\dot{e}}_{3} & = & -2b_{12}\sqrt{1-P_{r2}^{2}}\sin\varphi_{r2}+u_{3}-\alpha \left(t\right)\left(-2b_{02}\sqrt{1-P_{2}^{2}}\sin\varphi_{2}\right)-\dot{\alpha }\left(t\right)P_{2}\\ {\dot{e}}_{4} & = & -\omega_{12}\left(P_{r2}-P_{r1}-P_{r3}\right)+2b_{12}\frac{P_{r2}}{\sqrt{1-P_{r2}^{2}}}\cos\varphi_{r2}+u_{4}\\ & & -\alpha \left(t\right)\left[-\omega_{02}\left(P_{2}-P_{1}-P_{3}\right)+2b_{02}\frac{P_{2}}{\sqrt{1-P_{2}^{2}}}\cos\varphi_{2}\right]-\dot{\alpha }\left(t\right)\varphi_{2}\\ {\dot{e}}_{5} & = & -2b_{13}\sqrt{1-P_{r3}^{2}}\sin\varphi_{r3}+u_{5}-\alpha \left(t\right)\left(-2b_{03}\sqrt{1-P_{3}^{2}}\sin\varphi_{3}\right)-\dot{\alpha }\left(t\right)P_{3}\\ {\dot{e}}_{6} & = & -\omega_{13}\left(P_{r3}-P_{r1}-P_{r2}\right)+2b_{13}\frac{P_{r3}}{\sqrt{1-P_{r3}^{2}}}\cos\varphi_{r3}+u_{6}\\ & & -\alpha \left(t\right)\left[-\omega_{03}\left(P_{3}-P_{1}-P_{2}\right)+2b_{03}\frac{P_{3}}{\sqrt{1-P_{3}^{2}}}\cos\varphi_{3}\right]-\dot{\alpha }\left(t\right)\varphi_{3} \end{array}$$ (7)

We design the control law *u*_*i*_(*i* = 1, 2, 3, 4, 5, 6) as Eq (8) to make the synchronization errors *e*_1_, *e*_2_, *e*_3_, *e*_4_, *e*_5_, and *e*_6_ to stabilize at the origin.

$$\begin{array}{ccc} u_{1} & = & 2b_{11}\left[\sqrt{1-P_{r1}^{2}}\sin\varphi_{r1}-\alpha \left(t\right)\sqrt{1-P_{1}^{2}}\sin\varphi_{1}\right]+\dot{\alpha }\left(t\right)P_{1}-k_{1}e_{1}\\ u_{2} & = & \omega_{11}\left[\left(P_{r1}-P_{r2}-P_{r3}\right)-\alpha \left(t\right)\left(P_{1}-P_{2}-P_{3}\right)\right]\\ & & -2b_{11}\left[\frac{P_{r1}}{\sqrt{1-P_{r1}^{2}}}\cos\varphi_{r1}-\alpha \left(t\right)\frac{P_{1}}{\sqrt{1-P_{1}^{2}}}\cos\varphi_{1}\right]+\dot{\alpha }\left(t\right)\varphi_{1}-k_{2}e_{2}\\ u_{3} & = & 2b_{12}\left[\sqrt{1-P_{r2}^{2}}\sin\varphi_{r2}-\alpha \left(t\right)\sqrt{1-P_{2}^{2}}\sin\varphi_{2}\right]+\dot{\alpha }\left(t\right)P_{2}-k_{3}e_{3}\\ u_{4} & = & \omega_{12}\left[\left(P_{r2}-P_{r1}-P_{r3}\right)-\alpha \left(t\right)\left(P_{2}-P_{1}-P_{3}\right)\right]\\ & & -2b_{12}\left[\frac{P_{r2}}{\sqrt{1-P_{r2}^{2}}}\cos\varphi_{r2}-\alpha \left(t\right)\frac{P_{2}}{\sqrt{1-P_{2}^{2}}}\cos\varphi_{2}\right]+\dot{\alpha }\left(t\right)\varphi_{2}-k_{4}e_{4}\\ u_{5} & = & 2b_{13}\left[\sqrt{1-P_{r3}^{2}}\sin\varphi_{r3}-\alpha \left(t\right)\sqrt{1-P_{3}^{2}}\sin\varphi_{3}\right]+\dot{\alpha }\left(t\right)P_{3}-k_{5}e_{5}\\ u_{6} & = & \omega_{13}\left[\left(P_{r3}-P_{r1}-P_{r2}\right)-\alpha \left(t\right)\left(P_{3}-P_{1}-P_{2}\right)\right]\\ & & -2b_{13}\left[\frac{P_{r3}}{\sqrt{1-P_{r3}^{2}}}\cos\varphi_{r3}-\alpha \left(t\right)\frac{P_{3}}{\sqrt{1-P_{3}^{2}}}\cos\varphi_{3}\right]+\dot{\alpha }\left(t\right)\varphi_{3}-k_{6}e_{6} \end{array}$$ (8)

Furthermore, the update rule for the six unknown parameters *b*_11_, *b*_12_, *b*_13_, *ω*_11_, *ω*_12_, and *ω*_13_ are Eq (9) defined as follows:

$$\begin{array}{ccc} {\dot{b}}_{11} & = & 2\alpha \left(t\right)\sqrt{1-P_{1}^{2}}\sin\phi_{1}e_{1}-2\alpha \left(t\right)\frac{P_{1}}{\sqrt{1-P_{1}^{2}}}\cos\phi_{1}e_{2}-k_{7}e_{a}\\ {\dot{\omega }}_{11} & = & \alpha \left(t\right)\left(P_{1}-P_{2}-P_{3}\right)e_{2}-k_{8}e_{b}\\ {\dot{b}}_{12} & = & 2\alpha \left(t\right)\sqrt{1-P_{2}^{2}}\sin\phi_{2}e_{3}-2\alpha \left(t\right)\frac{P_{2}}{\sqrt{1-P_{2}^{2}}}\cos\phi_{2}e_{4}-k_{9}e_{c}\\ {\dot{\omega }}_{12} & = & \alpha \left(t\right)\left(P_{2}-P_{1}-P_{3}\right)e_{4}-k_{10}e_{d}\\ {\dot{b}}_{13} & = & 2\alpha \left(t\right)\sqrt{1-P_{3}^{2}}\sin\phi_{3}e_{5}-2\alpha \left(t\right)\frac{P_{3}}{\sqrt{1-P_{3}^{2}}}\cos\phi_{3}e_{6}-k_{11}e_{e}\\ {\dot{\omega }}_{13} & = & \alpha \left(t\right)\left(P_{3}-P_{1}-P_{2}\right)e_{6}-k_{12}e_{f} \end{array}$$ (9)

Where *k*_*i*_ > 0(*i* = 1, 2, 3, …, 12), and *e*_*a*_ = *b*_11_−*b*_01_, *e*_*b*_ = *ω*_11_ − *ω*_01_, *e*_*c*_ = *b*_12_ − *b*_02_, *e*_*d*_ = *ω*_12_ − *ω*_02_, *e*_*e*_ = *b*_13_ − *b*_03_, *e*_*f*_ = *ω*_13_ − *ω*_03_.

**Theorem.** For a given nonzero scaling function factor *α*(*t*), it can make response system(5) and drive system(4) to synchronize by the control law Eq (8) and the update rule Eq (9).

**Proof.** Choose the following Lyapunov function:

$$\begin{array}{c} V=\frac{1}{2}\left(e_{1}^{2}+e_{2}^{2}+e_{3}^{2}+e_{4}^{2}+e_{5}^{2}+e_{6}^{2}+e_{a}^{2}+e_{b}^{2}+e_{c}^{2}+e_{d}^{2}+e_{e}^{2}+e_{f}^{2}\right) \end{array}$$

The time derivative of V along the trajectory of the error system(6) is

$$\begin{array}{c} \dot{V}=\left(e_{1}{\dot{e}}_{1}+e_{2}{\dot{e}}_{2}+e_{3}{\dot{e}}_{3}+e_{4}{\dot{e}}_{4}+e_{5}{\dot{e}}_{5}+e_{6}{\dot{e}}_{6}+e_{a}{\dot{e}}_{a}+e_{b}{\dot{e}}_{b}+e_{c}{\dot{e}}_{c}+e_{d}{\dot{e}}_{d}+e_{e}{\dot{e}}_{e}+e_{f}{\dot{e}}_{f}\right), \end{array}$$

$$\begin{array}{ccc} \dot{V} & = & e_{1}\left[-2\left(b_{11}-b_{01}\right)\alpha \left(t\right)\sqrt{1-P_{1}^{2}}\sin\phi_{1}-k_{1}e_{1}\right]\\ & & +e_{2}\left[-\left(\omega_{11}-\omega_{01}\right)\alpha \left(t\right)\left(P_{1}-P_{2}-P_{3}\right)+2\left(b_{11}-b_{01}\right)\alpha \left(t\right)\frac{P_{1}}{\sqrt{1-P_{1}^{2}}}\cos\phi_{1}-k_{2}e_{2}\right]\\ & & +e_{3}\left[-2\left(b_{12}-b_{02}\right)\alpha \left(t\right)\sqrt{1-P_{2}^{2}}\sin\phi_{2}-k_{3}e_{3}\right]\\ & & +e_{4}\left[-\left(\omega_{12}-\omega_{02}\right)\alpha \left(t\right)\left(P_{2}-P_{1}-P_{3}\right)+2\left(b_{12}-b_{02}\right)\alpha \left(t\right)\frac{P_{2}}{\sqrt{1-P_{2}^{2}}}\cos\phi_{2}-k_{4}e_{4}\right]\\ & & +e_{5}\left[-2\left(b_{13}-b_{03}\right)\alpha \left(t\right)\sqrt{1-P_{3}^{2}}\sin\phi_{3}-k_{5}e_{5}\right]\\ & & +e_{6}\left[-\left(\omega_{13}-\omega_{03}\right)\alpha \left(t\right)\left(P_{3}-P_{1}-P_{2}\right)+2\left(b_{13}-b_{03}\right)\alpha \left(t\right)\frac{P_{3}}{\sqrt{1-P_{3}^{2}}}\cos\phi_{3}-k_{6}e_{6}\right]\\ & & +e_{a}\left[2\alpha \left(t\right)\sqrt{1-P_{1}^{2}}\sin\phi_{1}e_{1}-2\alpha \left(t\right)\frac{P_{1}}{\sqrt{1-P_{1}^{2}}}\cos\phi_{1}e_{2}-k_{7}e_{a}\right]\\ & & +e_{b}\left[\alpha \left(t\right)\left(P_{1}-P_{2}-P_{3}\right)e_{2}-k_{8}e_{b}\right]\\ & & +e_{c}\left[2\alpha \left(t\right)\sqrt{1-P_{2}^{2}}\sin\phi_{2}e_{3}-2\alpha \left(t\right)\frac{P_{2}}{\sqrt{1-P_{2}^{2}}}\cos\phi_{2}e_{4}-k_{9}e_{c}\right]\\ & & +e_{d}\left[\alpha \left(t\right)\left(P_{2}-P_{1}-P_{3}\right)e_{4}-k_{10}e_{d}\right]\\ & & +e_{e}\left[2\alpha \left(t\right)\sqrt{1-P_{3}^{2}}\sin\phi_{3}e_{5}-2\alpha \left(t\right)\frac{P_{3}}{\sqrt{1-P_{3}^{2}}}\cos\phi_{3}e_{6}-k_{11}e_{e}\right]\\ & & +e_{f}\left[\alpha \left(t\right)\left(P_{3}-P_{1}-P_{2}\right)e_{6}-k_{12}e_{f}\right]\\ & = & -k_{1}e_{1}^{2}-k_{2}e_{1}^{2}-k_{3}e_{3}^{2}-k_{4}e_{4}^{2}-k_{5}e_{5}^{2}-k_{6}e_{6}^{2}-k_{7}e_{a}^{2}-k_{8}e_{b}^{2}-k_{9}e_{c}^{2}-k_{10}e_{d}^{2}\\ & & -k_{11}e_{e}^{2}-k_{12}e_{f}^{2}\\ & = & -e^{T}Ke \end{array}$$

where *e* = (*e*_1_, *e*_2_, *e*_3_, *e*_4_, *e*_5_, *e*_6_, *e*_*a*_, *e*_*b*_, *e*_*c*_, *e*_*d*_, *e*_*e*_, *e*_*f*_)^*T*^, and *K* = *diag*(*k*_1_, *k*_2_, *k*_3_, *k*_4_, *k*_5_, *k*_6_, *k*_7_, *k*_8_, *k*_9_, *k*_10_, *k*_11_, *k*_12_)^*T*^

Because $\dot{V}\leq 0\;$, we have *e*_1_, *e*_2_, *e*_3_, *e*_4_, *e*_5_, *e*_6_, *e*_*a*_, *e*_*b*_, *e*_*c*_, *e*_*d*_, *e*_*e*_, *e*_*f*_ → 0 as *t* → ∞. i.e.,$\lim_{t\to \infty }\left||e|\right|=0$. Proof completed.

Simulation is performed in order to evaluate the feasibly and effectiveness of the proposed control law and the update rule for the 3-cell QCNN and 6^*th*^-order CNN synchronization method. The initial values and control parameters of the drive and the response system for a time-step of 0.1 are shown in Table 1.

**Table 1:** Initial values and control parameters of drive and response system.

Drive system	Response system	Control parameters of Response system
*x*_1_(0) = −0.92	*P*_*r*1_(0) = 0.1901	*b*_11_ = 0.5
*x*_2_(0) = 1.41	*ϕ*_*r*1_(0) = −184.3	*ω*_11_ = 0.6
*x*_3_(0) = −1.53	*P*_*r*2_(0) = 0.123	*b*_12_ = 0.4
*x*_4_(0) = 0.48	*ϕ*_*r*2_(0) = −147.3	*ω*_12_ = 0.7
*x*_5_(0) = 0.37	*P*_*r*3_(0) = 0.113	*b*_13_ = 0.7
*x*_6_(0) = −1.21	*ϕ*_*r*3_(0) = −197.85	*ω*_13_ = 0.5

In addition, the scaling function *α*(*t*) = 0.5 + 0.1 sin(*t*) and the control gains are defined as (*k*_1_, *k*_2_, *k*_3_, *k*_4_, *k*_5_, *k*_6_, *k*_7_, *k*_8_, *k*_9_, *k*_10_, *k*_11_, *k*_12_) = (1, 1, 1, 1, 1, 1, 1, 1, 1, 1, 1, 1).

**Fig 4:**
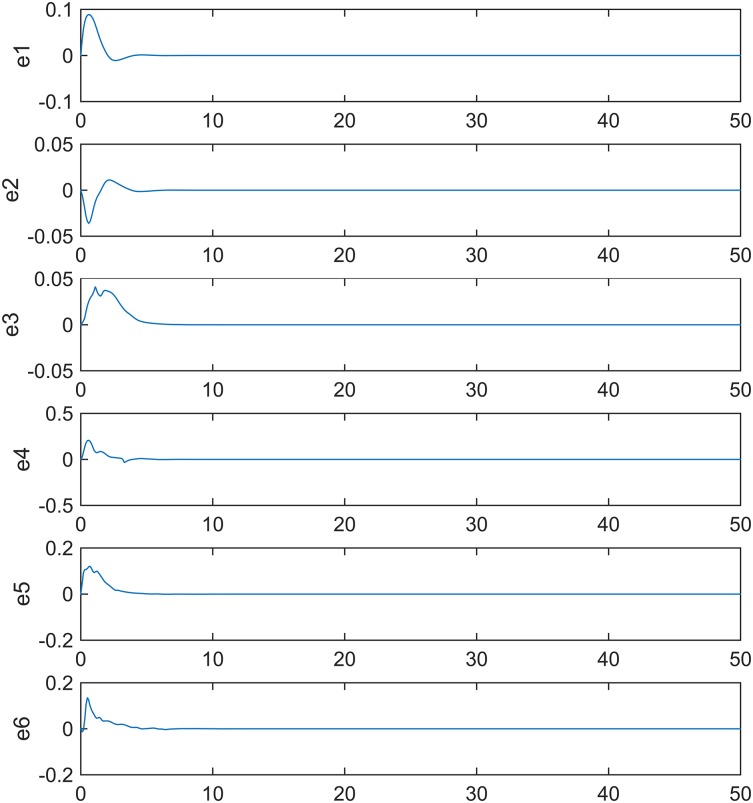
Error signals between the drive and the response system.

**Fig 5:**
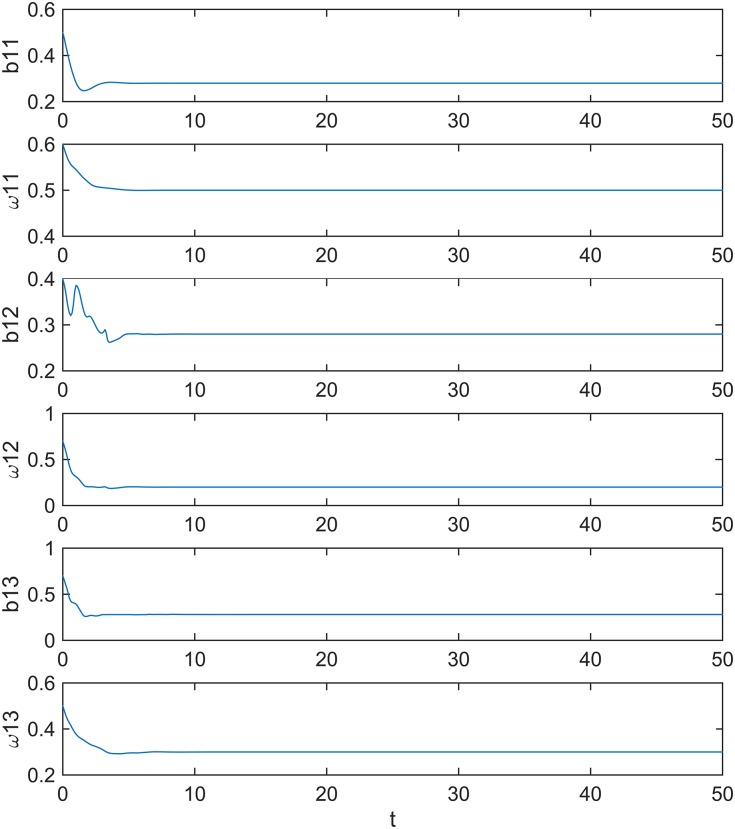
Estimated values for unknown parameters.

The simulations results are illustrated in Figs 4 and 5. Fig 4 shows that the errors *e*_1_, *e*_2_, *e*_3_, *e*_4_, *e*_5_, and *e*_6_ approach zero. Fig 5 shows that the estimated unknown parameters converge to *b*_11_ → 0.28, *ω*_11_ → 0.4, *b*_12_ → 0.28, *ω*_12_ → 0.35, *b*_13_ → 0.28, and *ω*_13_ → 0.25 as *t* → ∞. When t = 10, synchronization errors close 0 and unknown control parameters reach stability, which shows that the synchronization method is efficient.

## The semi-symmetric image encryption scheme

**Fig 6:**
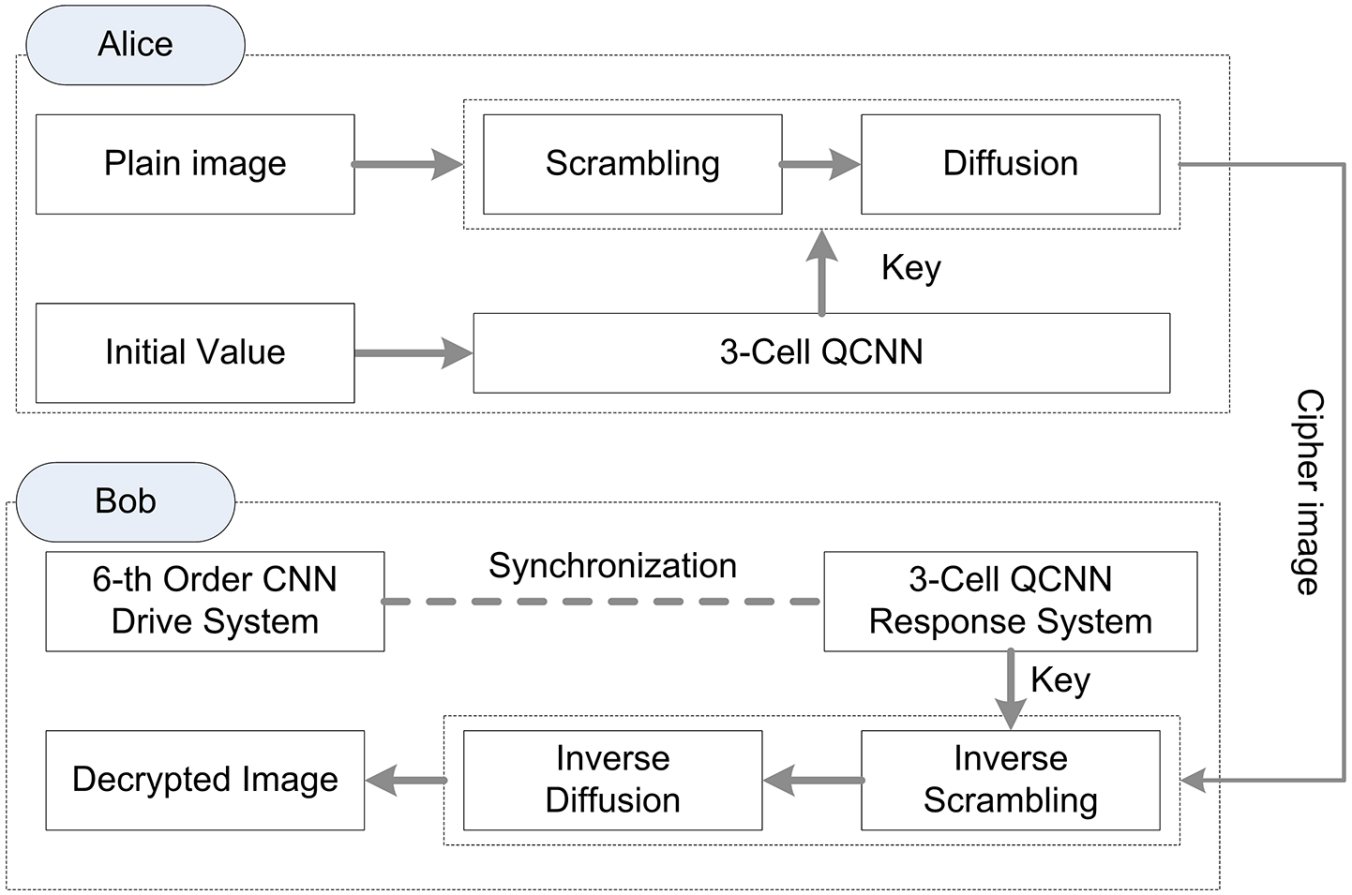
Semi-symmetric image encryption/decryption scheme.

In this paper, we propose a semi-symmetric image encryption/decryption scheme based on the function projective synchronization. The proposed scheme is illustrated in Fig 6. The scheme is deployed at the ends of Alice and Bob, respectively. Firstly, Alice adopts system(2) with initial parameters and control parameters to obtain the key. Bob adopts system(5) to obtain the key independently. Function projective synchronization ensures that Alice and Bob get the equivalent key. Secondly, Alice encrypts the plain image by his key and transmits the cipher image to Bob. Thirdly, Bob decrypts the cipher image with his key.

The proposed scheme is different with symmetric algorithms that Alice and Bob use in different key generation systems. The symmetric algorithms transmit the key by some extra security methods. The proposed scheme is similar to asymmetric algorithms that the keys generated by the two systems need not transmit to each other over other security link, which prevents security key leakage during the transmission.

Our scheme is a hybrid chaotic encryption algorithm. It consists of a scrambling stage and a diffusion stage. In encryption phase, 3-cell QCNN system(2) is used for scrambling and diffusing the plain image. In decryption phase, since the function projective synchronization is used to synchronize the response system(5) and drive system(4), the 6^*th*^-order CNN drive system(4) with control laws(8) and update rules(9) generates the key to decrypt the cipher image.

### Encryption algorithm

**Fig 7:**
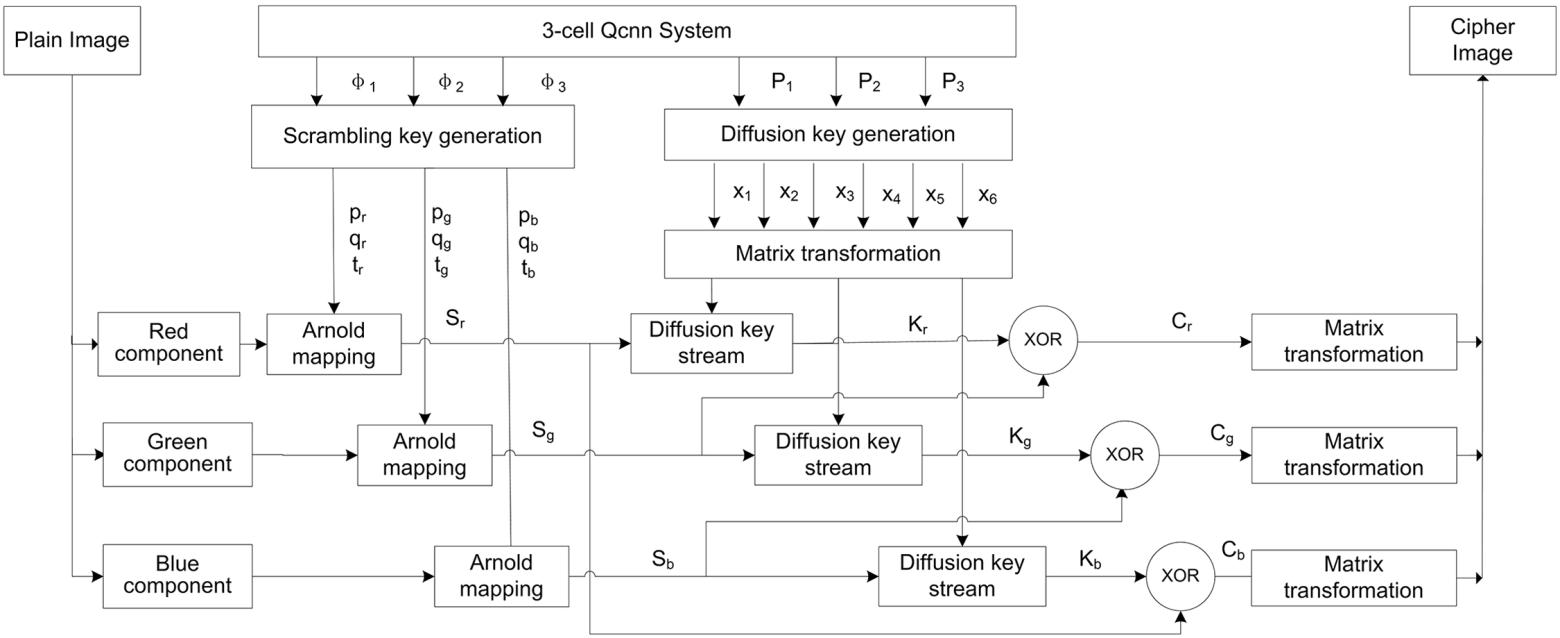
Encryption flowchart.

This encryption flowchart is presented in Fig 7.

The 3-cell QCNN system(2) is the encryption key generator. The initial conditions *ϕ*_1_(0), *ϕ*_2_(0), *ϕ*_3_(0), *P*_1_(0), *P*_2_(0), and *P*_3_(0) and control parameters *b*_01_, *b*_02_, *b*_03_, *ω*_01_, *ω*_02_, and *ω*_03_ are used to iterate system(2) M times. The results are *ϕ*_1_, *ϕ*_1_, *ϕ*_3_, *P*_1_, *P*_2_, and *P*_3_ encryption keys. In the scrambling stage, the Arnold mapping [37] defined that Eq (10) is used to scramble the three color components of the plain color image.

$$\begin{array}{c} \left(\begin{array}{c} x_{n+1}\\ y_{n+1} \end{array}\right)=A\left(\begin{array}{c} x_{n}\\ y_{n} \end{array}\right)\text{mod}\left(N\right)=\left[\begin{array}{cc} 1 & p\\ q & pq+1 \end{array}\right]\left(\begin{array}{c} x_{n}\\ y_{n} \end{array}\right)\text{mod}\left(N\right) \end{array}$$ (10)

Since det(*A*) = 1, the parameters are described as follows:

$$\begin{array}{ccc} p_{r} & = & floor(\text{mod}\left(\phi_{1}\times 2^{24}\right),N)\\ q_{r} & = & floor(\text{mod}\left(\text{mod}\left(\phi_{1}\times 2^{48}\right),2^{24}\right),N)\\ p_{g} & = & floor(\text{mod}\left(\phi_{2}\times 2^{24}\right),N)\\ q_{g} & = & floor(\text{mod}\left(\text{mod}\left(\phi_{2}\times 2^{48}\right),2^{24}\right),N)\\ p_{b} & = & floor(\text{mod}\left(\phi_{3}\times 2^{24}\right),N)\\ q_{b} & = & floor(\text{mod}\left(\text{mod}\left(\phi_{3}\times 2^{48}\right),2^{24}\right),N) \end{array}$$

The iterations of Arnold mappings are

$$\begin{array}{cc} t_{j}=floor\left(\left(\left(\text{mod}\left(\phi_{j}\times 2^{24}\right)+\text{mod}\left(\phi_{j}\times 2^{48}\right)\right),2^{24}\right),N\right) & j\in \{r,g,b\} \end{array}$$ (11)

The plain image is scrambled by Eq (10) in order to generate the permutation image. It is transformed into three 1 × (*N* × *N*) streams *S*_*j*_ = {*S*_*j*_(1), *S*_*j*_(2), ……*S*_*j*_(*N* × *N*)}, *j* ∈ {*r*, *g*, *b*} by arranging its pixels from top to bottom and left to right.

In the diffusion stage, 6^*th*^-order CNN system(4) is used to diffuse the image, which changes the permutation image pixel’s values. The initial conditions are described as follows:

$$\begin{array}{c} x_{i}\left(0\right)=\gamma_{i}P_{j},\;\left(i=1,2,3,4,5,6\;j=1,2,3\right) \end{array}$$

Of these initial conditions, *γ*_*i*_ is taken as the appropriate integer. *P*_*j*_ is chaotic value, so the initial conditions *x*_*i*_(0) is also chaotic value. Let the plain image be an N × N image.

The 6^*th*^-CNN is iterated $\frac{N\times N}{2}$ times and its result is divided into three matrices:*X*_*r*_, *X*_*g*_, and *X*_*b*_:

$$\begin{array}{ccc} X_{r} & = & \left[\begin{array}{cc} X_{1}\left(1\right) & X_{2}\left(1\right)\\ X_{1}\left(2\right) & X_{2}\left(2\right)\\ \vdots & \vdots \\ X_{1}\left(\frac{N\times N}{2}\right) & X_{2}\left(\frac{N\times N}{2}\right) \end{array}\right],X_{g}=\left[\begin{array}{cc} X_{3}\left(1\right) & X_{4}\left(1\right)\\ X_{3}\left(2\right) & X_{4}\left(2\right)\\ \vdots & \vdots \\ X_{3}\left(\frac{N\times N}{2}\right) & X_{4}\left(\frac{N\times N}{2}\right) \end{array}\right],\\ X_{b} & = & [\begin{array}{cc} X_{5}\left(1\right) & X_{6}\left(1\right)\\ X_{5}\left(2\right) & X_{6}\left(2\right)\\ \vdots & \vdots \\ X_{5}\left(\frac{N\times N}{2}\right) & X_{6}\left(\frac{N\times N}{2}\right) \end{array}]. \end{array}$$

Arranging matrix elements from top to bottom and from left to right, *X*_*r*_, *X*_*g*_, and *X*_*b*_ are transformed into three 1 × (*N* × *N*) streams:

$$\begin{array}{c} X_{j}\_Stream\left(i\right),\;\left(i=1,2,...,N\times N\;j\in \left\{r,g,b\right\}\right) \end{array}$$

The diffusion key streams, *K*_*j*_, are generated by using sequences *X*_*j*__*Stream* and *S*_*j*_ as described by Eq (12):

$$\begin{array}{ccc} K_{j}\left(i\right) & = & \text{mod}\{round[\left(abs\left(X_{j}\_Stream\left(i\right)\right)-floor\left(abs\left(X_{j}\_Stream\left(i\right)\right)\right)\right)\times 10^{14}\\ & & +S_{j}\left(i-1\right)],N\}\;\;i=1,2,......,N\times N,j\in \left\{r,g,b\right\} \end{array}$$ (12)

Let *S*_*j*_(0) = 127. The scramble image is shifted to the cipher image by key streams, *K*_*j*_.

$$\begin{array}{c} \left\{ \begin{array}{c} C_{r}\left(i\right)=bitxor\left(S_{g}\left(i\right),K_{r}\left(i\right)\right)\\ C_{g}\left(i\right)=bitxor\left(S_{b}\left(i\right),K_{g}\left(i\right)\right)\\ C_{b}\left(i\right)=bitxor\left(S_{r}\left(i\right),K_{b}\left(i\right)\right) \end{array}\right. \end{array}$$

*i* = 1, 2, ……, *N* × *N*, bitxor(.) function returns the bitwise exclusive OR value of two integers.

These *C*_*r*_, *C*_*g*_, and *C*_*b*_ row vectors are transformed into *N* × *N* matrix. Compose the three color components to obtain the encrypted image.

### Decryption algorithm

**Fig 8:**
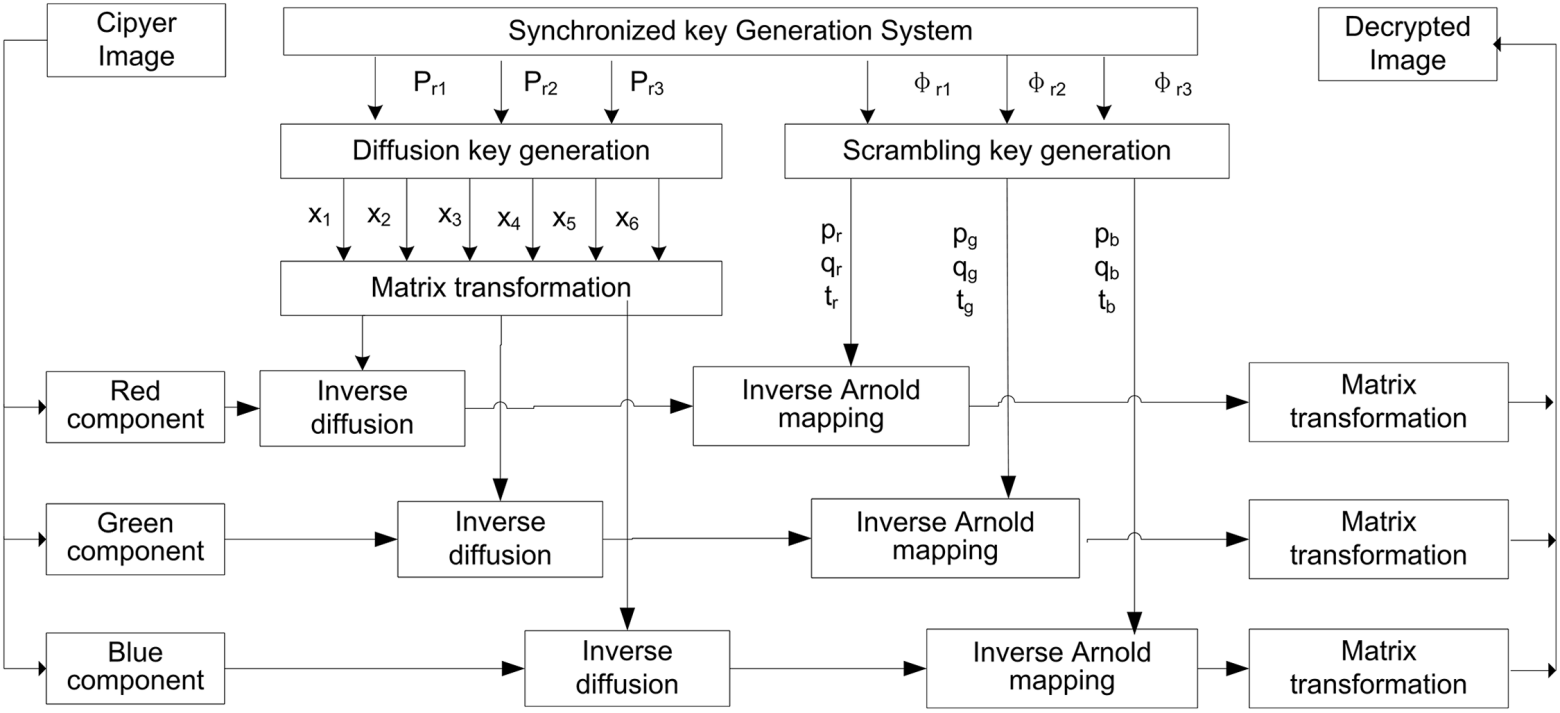
Decryption flowchart.

As shown in Fig 8, the decryption is the inverse process of the encryption, except that the decryption key *P*_*r*1_, *P*_*r*2_, *P*_*r*3_, *ϕ*_*r*1_, *ϕ*_*r*2_, and *ϕ*_*r*3_ are generated by the synchronized key generation system instead of 3-cell QCNN(2).

## Performance analysis

In this section, we perform 11 experiments to validate the proposed scheme. The results show that our scheme has good encryption performance.

### Key space analysis

The key space size is the total number of different keys that can be applied in the encryption process. The key space must be large enough to make brute-attacks infeasible. Stinson DR. [38] suggested that the key space should be at least 2^100^ to ensure a high level security. In our algorithm there are twelve parameters for the keys: six initial conditions P_1_, Φ_1_, P_2_, Φ_2_, P_3_, Φ_3_ and six control parameters ${\text{b}}_{01},{\text{b}}_{02},{\text{b}}_{03},\omega_{01},\omega_{02},\omega_{03}$. They are all floating point numbers. According to the IEEE floating-point standard [39], the computational precision of the 64-bit double-precision numbers is 2^—52^. So the key space of the proposed encryption method is (2^52^)^12^ = 2^624^, which is sufficiently large enough to resist all kinds of brute-force attacks.

### Key sensitivity analysis

A secure encryption algorithm must be sensitivity to its keys which satisfies the requirement of resisting brute-force attack. Under the same experiment condition as Eq (13). *P*_1_(0), *P*_2_(0), *P*_3_(0), *ϕ*_1_(0), *ϕ*_2_(0), *ϕ*_3_(0) are QCNN system(2) initial conditions, used as user keys in the proposed encryption scheme.

$$\begin{array}{c} Key=\{\begin{array}{c} P_{1}\left(0\right)=-0.131;P_{2}\left(0\right)=-0.135;P_{3}\left(0\right)=-0.123;\\ \phi_{1}\left(0\right)=-184.9;\phi_{2}\left(0\right)=147.3414;\phi_{1}\left(0\right)=-196.852;\\ b_{01}=b_{01}=b_{01}=0.28;\\ \omega_{01}=0.5;\omega_{02}=0.2;\omega_{03}=0.3; \end{array}\} \end{array}$$ (13)

With a tiny difference in the encryption keys, six groups of test cases are designed, which differ 10^—13^ to every encryption key, respectively.

$$\begin{array}{ccc} Key1 & = & \left\{\begin{array}{c} P_{1}\left(0\right)=-0.131+10^{-13};P_{2}\left(0\right)=-0.135;P_{3}\left(0\right)=-0.123;\\ \phi_{1}\left(0\right)=-184.9;\phi_{2}\left(0\right)=147.3414;\phi_{1}\left(0\right)=-196.852;\\ b_{01}=b_{01}=b_{01}=0.28;\\ \omega_{01}=0.5;\omega_{02}=0.2;\omega_{03}=0.3; \end{array}\right\}\\ Key2 & = & \left\{\begin{array}{c} P_{1}\left(0\right)=-0.131;P_{2}\left(0\right)=-0.135+10^{-13};P_{3}\left(0\right)=-0.123;\\ \phi_{1}\left(0\right)=-184.9;\phi_{2}\left(0\right)=147.3414;\phi_{1}\left(0\right)=-196.852;\\ b_{01}=b_{01}=b_{01}=0.28;\\ \omega_{01}=0.5;\omega_{02}=0.2;\omega_{03}=0.3; \end{array}\right\}\\ Key3 & = & \left\{\begin{array}{c} P_{1}\left(0\right)=-0.131;P_{2}\left(0\right)=-0.135;P_{3}\left(0\right)=-0.123+10^{-13};\\ \phi_{1}\left(0\right)=-184.9;\phi_{2}\left(0\right)=147.3414;\phi_{1}\left(0\right)=-196.852;\\ b_{01}=b_{01}=b_{01}=0.28;\\ \omega_{01}=0.5;\omega_{02}=0.2;\omega_{03}=0.3; \end{array}\right\}\\ Key4 & = & \left\{\begin{array}{c} P_{1}\left(0\right)=-0.131;P_{2}\left(0\right)=-0.135;P_{3}\left(0\right)=-0.123;\\ \phi_{1}\left(0\right)=-184.9+10^{-13};\phi_{2}\left(0\right)=147.3414;\phi_{1}\left(0\right)=-196.852;\\ b_{01}=b_{01}=b_{01}=0.28;\\ \omega_{01}=0.5;\omega_{02}=0.2;\omega_{03}=0.3; \end{array}\right\}\\ Key5 & = & \left\{\begin{array}{c} P_{1}\left(0\right)=-0.131;P_{2}\left(0\right)=-0.135;P_{3}\left(0\right)=-0.123;\\ \phi_{1}\left(0\right)=-184.9;\phi_{2}\left(0\right)=147.3414+10^{-13};\phi_{1}\left(0\right)=-196.852;\\ b_{01}=b_{01}=b_{01}=0.28;\\ \omega_{01}=0.5;\omega_{02}=0.2;\omega_{03}=0.3; \end{array}\right\}\\ Key6 & = & \left\{\begin{array}{c} P_{1}\left(0\right)=-0.131;P_{2}\left(0\right)=-0.135;P_{3}\left(0\right)=-0.123;\\ \phi_{1}\left(0\right)=-184.9;\phi_{2}\left(0\right)=147.3414;\phi_{1}\left(0\right)=-196.852+10^{-13};\\ b_{01}=b_{01}=b_{01}=0.28;\\ \omega_{01}=0.5;\omega_{02}=0.2;\omega_{03}=0.3; \end{array}\right\} \end{array}$$

Table 2 lists the percentage of different pixels in RGB color component using Key or Key1, Key2, …, Key6 seven encrypt images, respectively. Therefore, it can be concluded the slightly deviation in the key brings out absolutely different in the corresponding encryption images. Consequently, the proposed scheme has a high key sensitivity and can resist the brute-force attack.

**Table 2:** Percentage of different pixels in RGB color component using Key or Key1, Key2, …, Key6 encrypted images.

Image color component	Key1	Key2	Key3	Key4	Key5	Key6
Airplane Red	99.5956	99.6155	99.5834	99.646	99.5895	99.588
Airplane Green	99.588	99.5911	99.5895	99.5911	99.5911	99.6262
Airplane Blue	99.5743	99.6033	99.6216	99.5941	99.6155	99.5941
Cablecar Red	99.6094	99.6185	99.6231	99.6002	99.6048	99.6353
Cablecar Green	99.5895	99.6017	99.5987	99.5926	99.6170	99.5941
Cablecar Blue	99.5865	99.5972	99.5926	99.6201	99.5880	99.5789
Cornfield Red	99.6567	99.6170	99.6307	99.5972	99.6002	99.5804
Cornfield Green	99.6109	99.6063	99.6109	99.6124	99.5804	99.6323
Cornfield Blue	99.6033	99.5850	99.5895	99.6277	99.5499	99.6063
Peppers Red	99.6124	99.5926	99.6246	99.6078	99.6124	99.6231
Peppers Green	99.6338	99.614	99.588	99.6231	99.5438	99.5804
Peppers Blue	99.6063	99.614	99.5636	99.6033	99.6429	99.5605
Boat Red	99.6017	99.5926	99.5911	99.6048	99.6078	99.588
Boat Green	99.6033	99.6155	99.6399	99.6658	99.5712	99.5834
Boat Blue	99.588	99.5834	99.6246	99.6109	99.588	99.5804
Fruits Red	99.5743	99.5804	99.6384	99.6460	99.6307	99.5865
Fruits Green	99.6201	99.6155	99.5834	99.6506	99.6490	99.5758
Fruits Blue	99.6414	99.6307	99.5712	99.6155	99.6140	99.5987
Yacht Red	99.5880	99.5850	99.6124	99.5911	99.6582	99.6078
Yacht Green	99.5728	99.6521	99.5911	99.6170	99.5758	99.6155
Yacht Blue	99.6521	99.5956	99.6155	99.6429	99.6307	99.5941

### Histogram analysis

**Fig 9:**
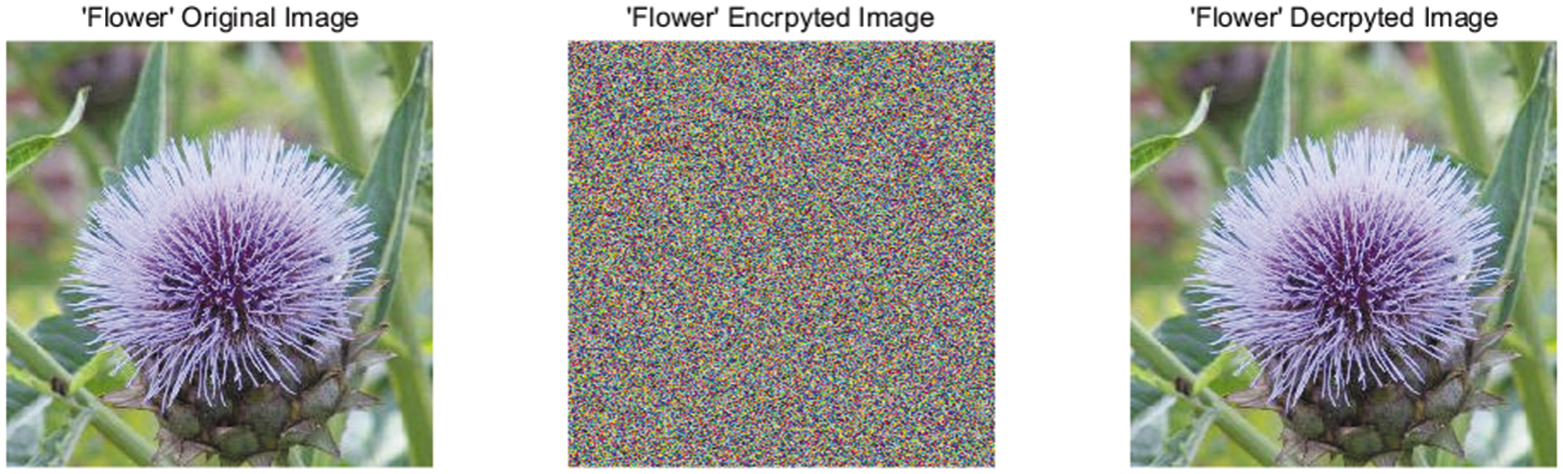
“Flower” original, cipher image and decrypted image.

**Fig 10:**
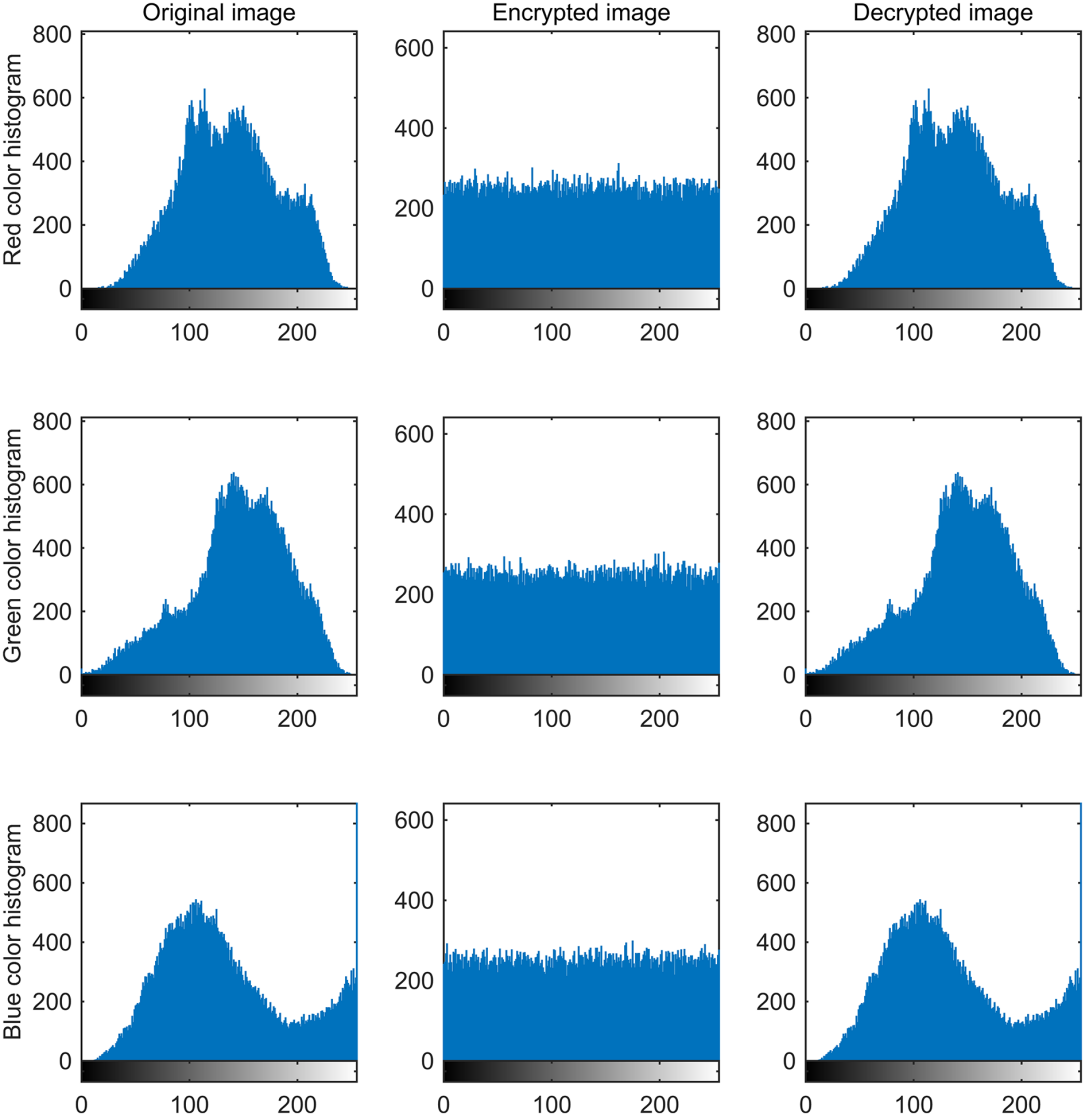
Three color component histograms of “Flower” original, encrypted and decrypted image.

**Fig 11:**
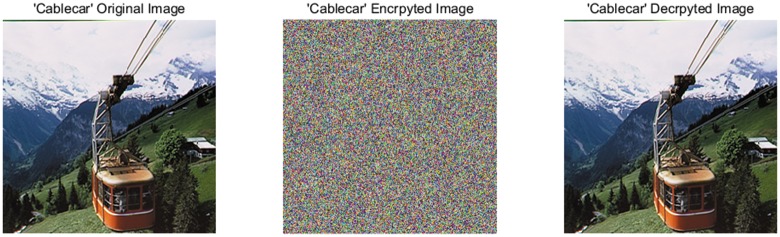
“Cablecar” original, encrypted and decrypted image.

**Fig 12:**
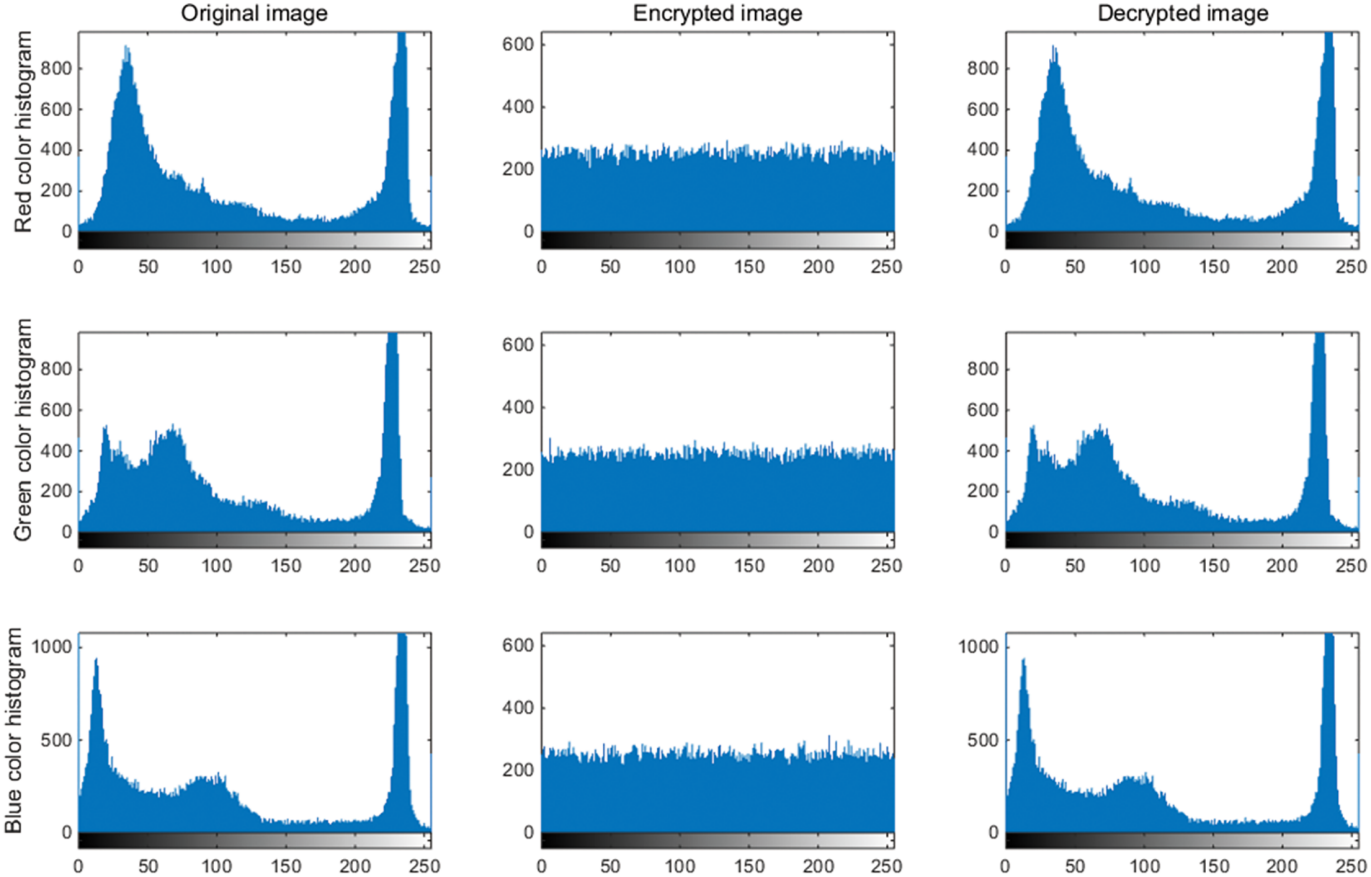
Three color component histograms of “Cablecar” original, encrypted and decrypted image.

A good image encryption approach should always generate the uniform histogram of cipher image for any plain image. The plain images, cipher images, decrypted images, and the histograms of their three-color components are shown in Figs 9–12. As illustrated, the histograms of the encrypted images are fairly uniform and significantly different from the respective histograms of the original images. Hence, our proposed scheme does not provide any clue to statistical attacks.

### Correlation coefficient analysis

To test the correlation of pixels (vertical, horizontal, diagonal), we randomly select 4000 adjacent pairs of the plain image and the cipher image, and calculated the correlation coefficients of pixels, according to the following formula:

$$\begin{array}{ccc} e(x) & = & \frac{1}{N}\sum_{i=1}^{N}x_{i}\\ d(x) & = & \frac{1}{N}\sum_{i=1}^{N}{\left(x_{i}-e\left(x\right)\right)}^{2}\\ \text{cov}(x,y) & = & \frac{1}{N}\sum_{i=1}^{N}\left(x_{i}-e\left(x\right)\right)\left(y_{i}-e\left(y\right)\right)\\ r_{xy} & = & \frac{\text{cov}(x,y)}{\sqrt{d(x)}\sqrt{d(y)}} \end{array}$$

**Fig 13:**
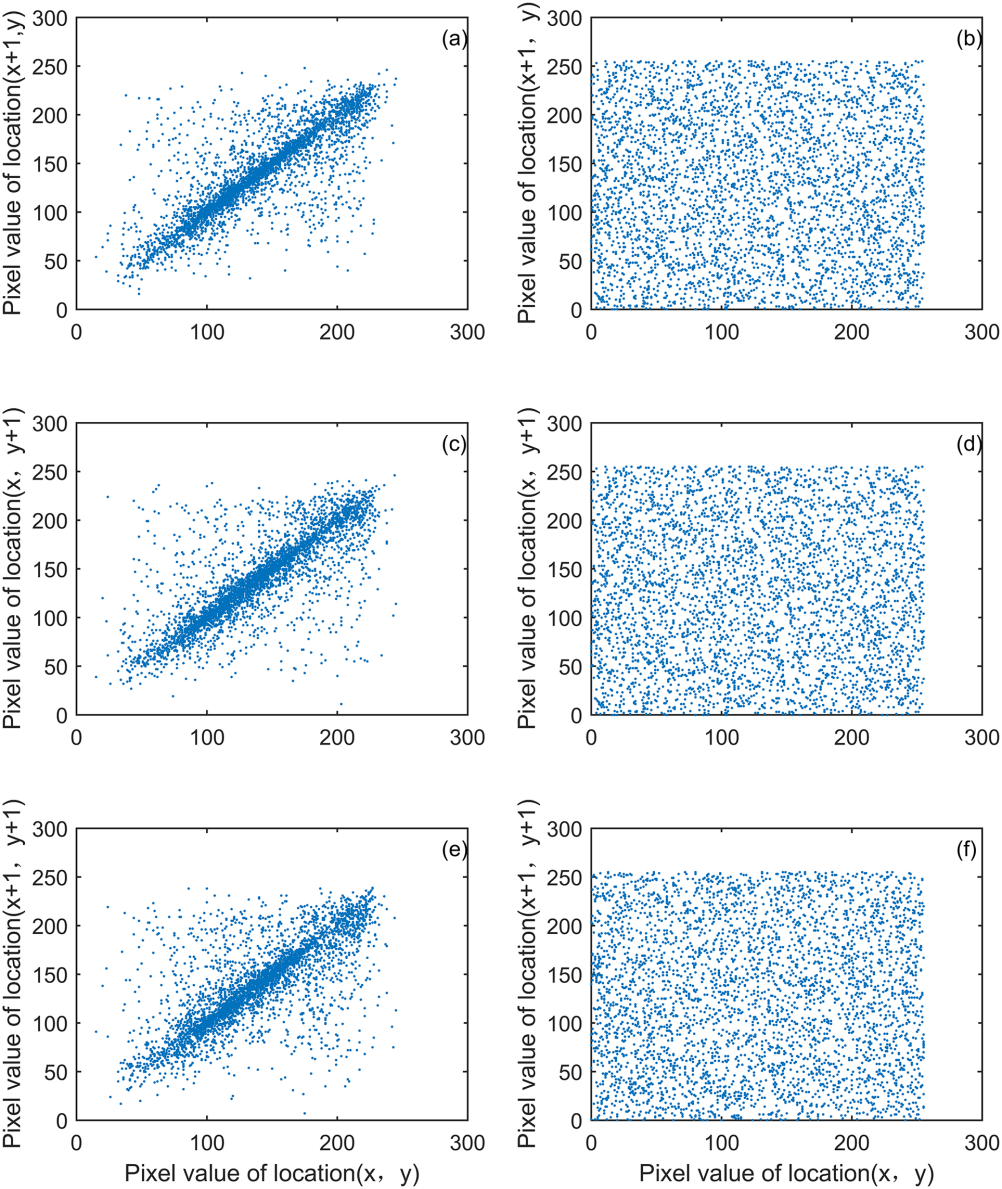
“Flower” image correlation of two adjacent pixels. (a) the distribution of two horizontal adjacent pixels in the original image, (b) the distribution of two horizontal adjacent pixels in the encryption image, (c) the distribution of two vertically adjacent pixels in the original image, (d) the distribution of two vertically adjacent pixels in the encryption image, (e) the distribution of two diagonally adjacent pixels in the original image, and (f) the distribution of two diagonally adjacent pixels in the encryption image.

**Fig 14:**
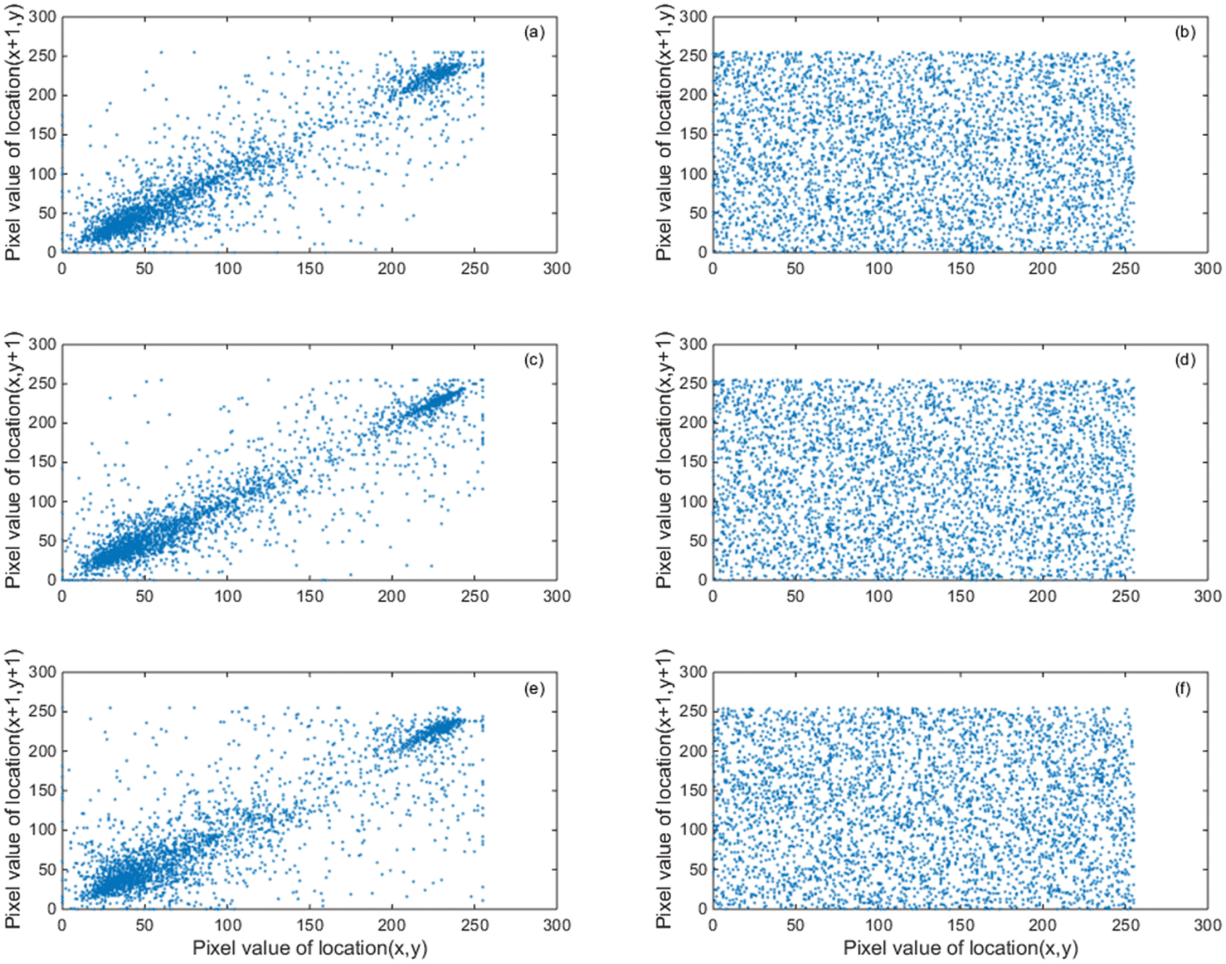
“Cablecar” image correlation of two adjacent pixels. (a) the distribution of two horizontal adjacent pixels in the original image, (b) the distribution of two horizontal adjacent pixels in the encryption image, (c) the distribution of two vertically adjacent pixels in the original image, (d) the distribution of two vertically adjacent pixels in the encryption image, (e) the distribution of two diagonally adjacent pixels in the original image and (f) the distribution of two diagonally adjacent pixels in the encryption image.

Figs 13 and 14 show image “Flower” and “Cablecar” correlation of two adjacent pixels. Table 3 provides more tests of the correlations, which show that two adjacent pixels in the plain images are highly correlated while the cipher images showed negligible correlations. The result indicates that our proposed encryption model functions properly.

**Table 3:** Correlation coefficients of original images and encryption images.

Encryption algorithm	Horizontal	Vertical	Diagonal
Ref [20] algorithm	0.0681	0.0845	-
Ref [22] algorithm	-0.0318	0.0965	0.0362
Ref [24] algorithm	0.0051	-0.0093	-0.0205
Ref [26] algorithm	0.0086	0.0195	-0.0093
Ref [40] algorithm	-0.00164	0.01304	-0.01911
Ref [41] algorithm	0.0773	0.0770	-0.0.0693
*Proposed algorithm “Flower”*	-0.0062	0.0052	0.0043
*Proposed algorithm “Cablecar”*	-0.0061	0.0070	0.0102

#### Information entropy analysis

Information entropy is thought to be one of the most important features of randomness. To measure the entropy, *H*(*m*), of a source *m*, the following equation can be employed:

$$H\left(m\right)=-\sum_{i=0}^{2^{n}-1}p\left(m_{i}\right)\log_{2}p\left(m_{i}\right)$$

where *p*(*m*_*i*_) represents the probability of symbol *m*_*i*_, and the entropy is expressed in bits. For example, when *n* = 8, the image color strength value is *m* = {*m*_0_, ……, *m*_255_}. For a random process, each symbol has equal probability, *p*(*m*_*i*_) = 1/256, *H*(*m*) = 8. In general, the entropy value of the message is smaller than 8 but should to be close to ideal. Table 4 provides a comparison of average entropy values for a considerable number of images for the proposed method and some other methods. We noticed that our scheme outperforms other schemes and approaches the ideal value of 8.

**Table 4:** Information entropy of ciphered images with three color components.

Encryption algorithm	red	green	blue
Ref [20] algorithm	7.9732	7.9750	7.9715
Ref [22] algorithm	7.9851	7.9852	7.9832
Ref [23] algorithm	7.9991	7.9990	7.9989
Ref [26] algorithm	7.9971	7.9968	7.9974
Ref [41] algorithm	7.9974	7.9966	7.9975
Ref [42] algorithm	7.9808	7.9811	7.9814
*Proposed algorithm “Flower”*	7.9993	7.9992	7.9987
*Proposed algorithm “Cablecar”*	7.9972	7.9975	7.9972

### Differential attack

Cryptanalysis features an important method called differential attack to crack the encryption algorithm in order to quantitatively measure the influence of a one–pixel change on the cipher image. This influence can be measured via the number of pixel change rate (NPCR) and the unified averaged changing intensity (UACI), which are computed with the following formula:

$$\begin{array}{ccc} NPCR & = & \frac{\sum_{i,j}D(i,j)}{W\times H}\times 100\%\\ UACI & = & \frac{1}{W\times H}\left(\sum_{i,j}\frac{|C\left(i,j\right)-C^{\prime }\left(i,j\right)|}{255}\right)\times 100\% \end{array}$$

where *W* and *H* represent the width and height of the image, respectively. *C*(*i*, *j*) and *C*′(*i*, *j*) are the ciphered images before and after one pixel of the plain image is changed. For position (*i*, *j*), if *C*(*i*, *j*) ≠ *C*′(*i*, *j*), then *D*(*i*, *j*) = 1; else *D*(*i*, *j*) = 0. We tested the NPCR and UACI values for images Flower and Cablecar for the proposed scheme. As shown in Tables 5 and 6, the proposed scheme is very sensitive with small changes in the plain image. This result shows that our scheme can resist differential attack well.

**Table 5:** NPCR of ciphered image.

NPCR	red	green	blue
Ref [23] algorithm	99.6239	99.6216	99.6236
Ref [27] algorithm	99.5445	99.5875	99.5374
Ref [29] algorithm	99.6155	99.6536	99.6475
Ref [41] algorithm	99.6399	99.6002	99.5773
Ref [42] algorithm	99.6170	99.6002	99.5925
*Proposed algorithm “Flower”*	99.6002	99.6063	99.5834
*Proposed algorithm “Cablecar”*	99.6140	99.6094	99.5926

**Table 6:** UACI of ciphered image.

UACI	red	green	blue
Ref [23]algorithm	33.6623	33.6827	33.6754
Ref [27]algorithm	34.3174	34.1786	33.6467
Ref [29]algorithm	33.6970	34.3251	32.2345
Ref [41]algorithm	33.5916	33.5010	33.4853
Ref [42]algorithm	33.4252	33.5898	33.4466
*Proposed algorithm “Flower”*	33.3635	33.4891	33.5000
*Proposed algorithm “Cablecar”*	33.4828	33.2790	33.4992

### Known plaintext attack and chosen plaintext attack

The diffusion key stream K_*j*_, in Eq (12), not only depends on the security key (initial conditions of 3-cell QCNN, *P*_1_(0), *P*_2_(0), *P*_3_(0), *ϕ*_1_(0), *ϕ*_2_(0), *ϕ*_3_(0)) but also on the plain image itself. Hence, when the same security key encrypts different images, the diffusion key streams are different. Therefor it is ineffective on input an all “0” or all “1” image into this scheme. Accordingly, our scheme can resist known plaintext attack and chosen plaintext attack.

### Encryption quality analysis

In an ideal cryptographic model, encrypted images should have uniform histogram distribution to hide pixels relevant information. It implies the encryption algorithm changes the the cipher pixel value to make the probability of each cipher pixel being totally uniform. Literature [43] gives a method for estimating the encryption quality, deviation from uniform histogram(*D*_*H*_), which is given by Eq (14).

$$\begin{array}{c} D_{H}=\frac{\sum_{C_{i}}^{255}\text{|}{\text{H}}_{C_{i}}\;\text{-}\;{\text{H}}_{C}\text{|}}{M\times N} \end{array}$$ (14)

In Eq (14), *M* × *N* is the image size and *C*_*i*_ is the image pixel gray or color level, *C*_*i*_ ∈ [0, 255]. H_*Ci*_ is the histogram value at index i, and H_*C*_ is the actual histogram of encrypted image. The smaller *D*_*H*_ value indicates the more uniform histogram distribution and the higher encryption quality.

We obtain *D*_*H*_ comparison reports for three images through using our algorithm with other chaotic encryption algorithms in Ref [25]. As can be seen from Table 7, all *D*_*H*_ values are very low. Moreover, our algorithm has more uniform histogram distribution and better encryption quality than Ref [10, 13, 25, 44].

**Table 7:** Deviation from uniform histogram(*D*_*H*_).

Image	Proposed algorithm	Ref [10]	Ref [13]	Ref [25]	Ref [44]
Peppers	0.0492	0.0938	0.0979	0.0917	0.0977
Airplane	0.0518	0.0969	0.0995	0.0983	0.0943
Boat	0.0524	0.0902	0.0995	0.0958	0.0985

### Chi-square test

A Chi-squared test [45, 46], also written as *X*^2^ test, is any statistical hypothesis test wherein the sampling distribution of the test statistic is a chi-squared distribution when the null hypothesis is true. Chi-squared test illustrate the possibility of statistical attacks. To evaluate if and what extent distribution of encrypted image histograms approach the features of a uniform distribution, Chi-squared tests are computed for 7 cipher images’ histograms, and then are summarized in Table 8. We find the histograms of the encrypted images are fairly uniform, so the proposed scheme can defend statistical attack.

**Table 8:** Chi-square test results for encrypted images.

Test Image	*X*^2^ P-value	Decision on H_0_
Cablecar	0.710	Accepted
Cornfield	0.969	Accepted
Peppers	0.791	Accepted
Airplane	0.321	Accepted
Fruits	0.580	Accepted
Boat	0.679	Accepted
Yacht	0.684	Accepted

### NIST SP800-22 test

NIST SP800-22 test [47] includes 16 test methods, which are used to analyse the randomness of binary sequences generated by cipher systems. We performed all the 16 tests for 65536–8 bits key stream sequence and the results are shown in Table 9. From the Table 9, it shows that our scheme goes through all NIST SP800-22 tests successfully. Therefore, the key stream sequence is absolutely random in our scheme.

**Table 9:** NIST SP800-22 tests results for encrypted key.

Test name		P-value	Result
Frequency		0.9801	Success
Block-frequency		0.2775	Success
Runs		0.3160	Success
Long runs of ones		0.3954	Success
Rank		0.0296	Success
Spectral DFT		0.1550	Success
No overlapping templates		0.9967	Success
Overlapping templates		0.4514	Success
Universal		0.6556	Success
Linear complexity		0.9056	Success
Serial	P-value1	0.9266	Success
Serial	P-value2	0.7865	Success
Approximate entropy		0.6375	Success
Cumulative sums forward		0.5436	Success
Cumulative sums reverse		0.5651	Success
Random excursions	X = -4	0.7220	Success
	X = -3	0.7752	Success
	X = -2	0.2677	Success
	X = -1	0.2656	Success
	X = 1	0.1007	Success
	X = 2	0.3482	Success
	X = 3	0.4977	Success
	X = 4	0.5168	Success
Random excursions variant	X = -9	0.2492	Success
	X = -8	0.1723	Success
	X = -7	0.2026	Success
	X = -6	0.4146	Success
	X = -5	0.4073	Success
	X = -4	0.3178	Success
	X = -3	0.3753	Success
	X = -2	0.6367	Success
	X = -1	0.4315	Success
	X = 1	0.6596	Success
	X = 2	0.7163	Success
	X = 3	0.6525	Success
	X = 4	0.4903	Success
	X = 5	0.3089	Success
	X = 6	0.2110	Success
	X = 7	0.1905	Success
	X = 8	0.1267	Success
	X = 9	0.1269	Success

### Encryption speed and computation complexity

The encryption speed is an important issue for a well applicable encryption system. Nevertheless, it depends on many factors as hardware, software and programming [25]. Ref [24, 48] have performed encryption speed tests for some algorithms in [5, 7, 24, 48–52] at the same enviorment. From Ref [48], we know that the encryption speed of algorithm [5, 7, 48, 49] are >10s, 2.3s, 1.25s, and 2.901s respectively. The execution time of scheme in [24, 50–52] are 155ms, 173ms, 2.089s and 334ms [24]. In our scheme, Arnold mapping iteration times *t*_*j*_ in Eq (11), is randomness for improving security, so it is hard to build a baseline to compare encryption speed with other methods, especially programming skill and code optimization [25]. So we give the encryption speed with different Arnold mapping iteration times in Table 10, and the environment is Microsoft Windows 7, Matlab8.4, a laptop with an Intel Xeon CPU E3-1220 v3 3.10GHz, 8.00GB RAM. As can be seen from the Table 10, our scheme has an acceptable speed.

**Table 10:** The speed range for the proposed algorithm.

Image	Iteration 1 time Speed(ms)	Iteration 10 times Speed(ms)	Iteration 20 times Speed(ms)	Iteration 30 times Speed(ms)	Iteration 40 times Speed(ms)	Iteration 50 times Speed(ms)
Peppers	103	368	666	960	1261	1554
Cablecar	102	359	644	931	1215	1501
Airplane	104	372	666	959	1256	1550
Cornfield	101	358	646	934	1214	1512
Boat	101	371	668	960	1256	1553
Fruits	102	363	655	931	1216	1528
Yacht	101	358	644	932	1215	1504

Additionally, the computation complexity relies on the number of operations and steps to fulfill the encryption. Our scheme needs O(n) to complete the entire encryption process, where n is the pixel number of images. Thus, the efficiency of the proposed algorithm is competent in the application level encryption requirements.

## Conclusion

In this paper, a semi-symmetric image encryption scheme based on function projective synchronization between two hyperchaotic systems is proposed, and it has several advantages such as great speed, relatively low complexity compared respectively to symmetric and asymmetric algorithms. Especially, the key is generated simultaneously in encryption side and decryption side independently, which effectively avoids the key transmission and threats of key exposure. The presented scheme is a hybrid chaotic encryption algorithm and it consists of a scrambling stage and a diffusion stage. Moreover, the 6^*th*^-order CNN is not only regarded as the drive system for the key synchronization, but also is used for diffusing key generation to enhance the security and sensitivity of the scheme. The simulation experiments and security performance analyses show that our scheme has a satisfactory security performance.

## Supporting information

S1 Fig “Flower” original image. (TIF)

S2 Fig “Cablecar” original image. (TIF)

S3 Fig “Airplane” original image. (TIF)

S4 Fig “Boat” original image. (TIF)

S5 Fig “Cornfield” original image. (TIF)

S6 Fig “Fruits” original image. (TIF)

S7 Fig “Peppers” original image. (TIF)

S8 Fig “Yacht” original image. (TIF)
