# Supplementary figures and images for: A semi-symmetric image encryption scheme based on the function projective synchronization of two hyperchaotic systems
Source: PLoS One. 2017 Sep 14;12(9):e0184586. doi: 10.1371/journal.pone.0184586 (PMC5599019; doi:10.1371/journal.pone.0184586)

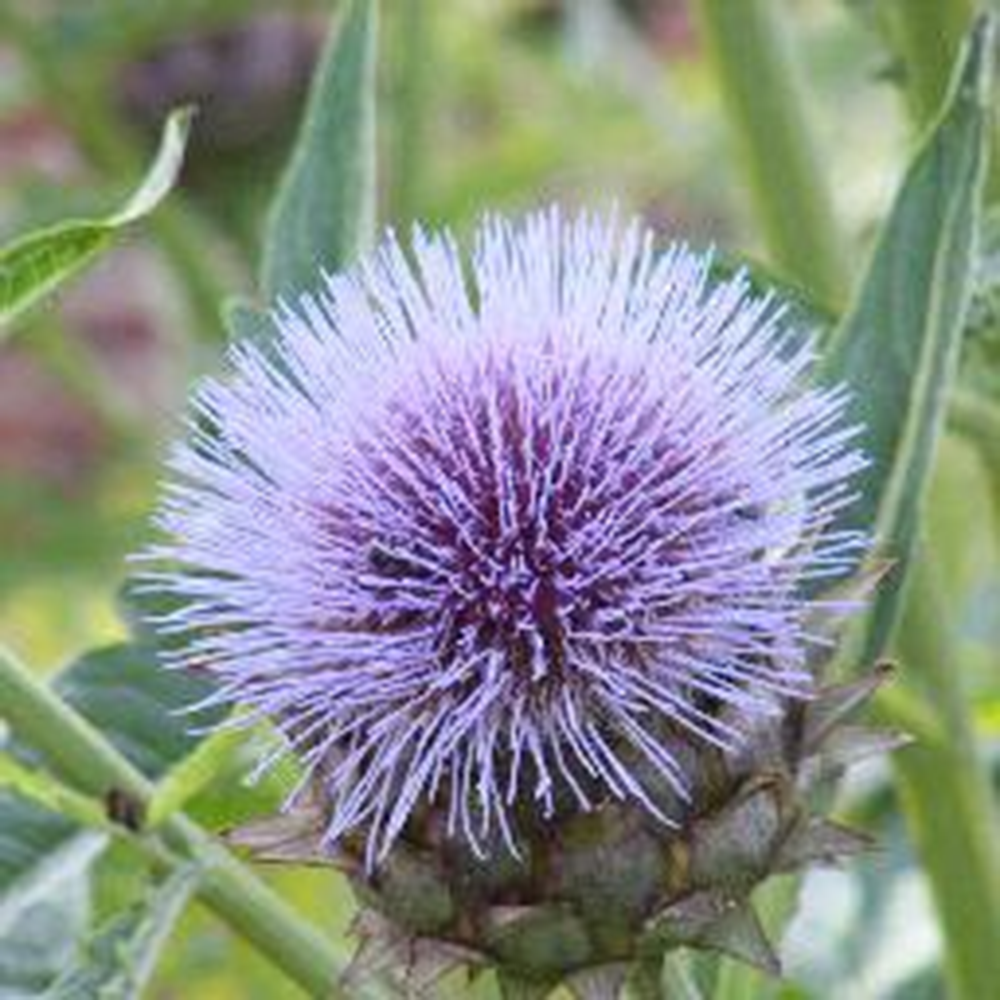

Supplement: S1 Fig — (TIF) [file pone.0184586.s001.tif]

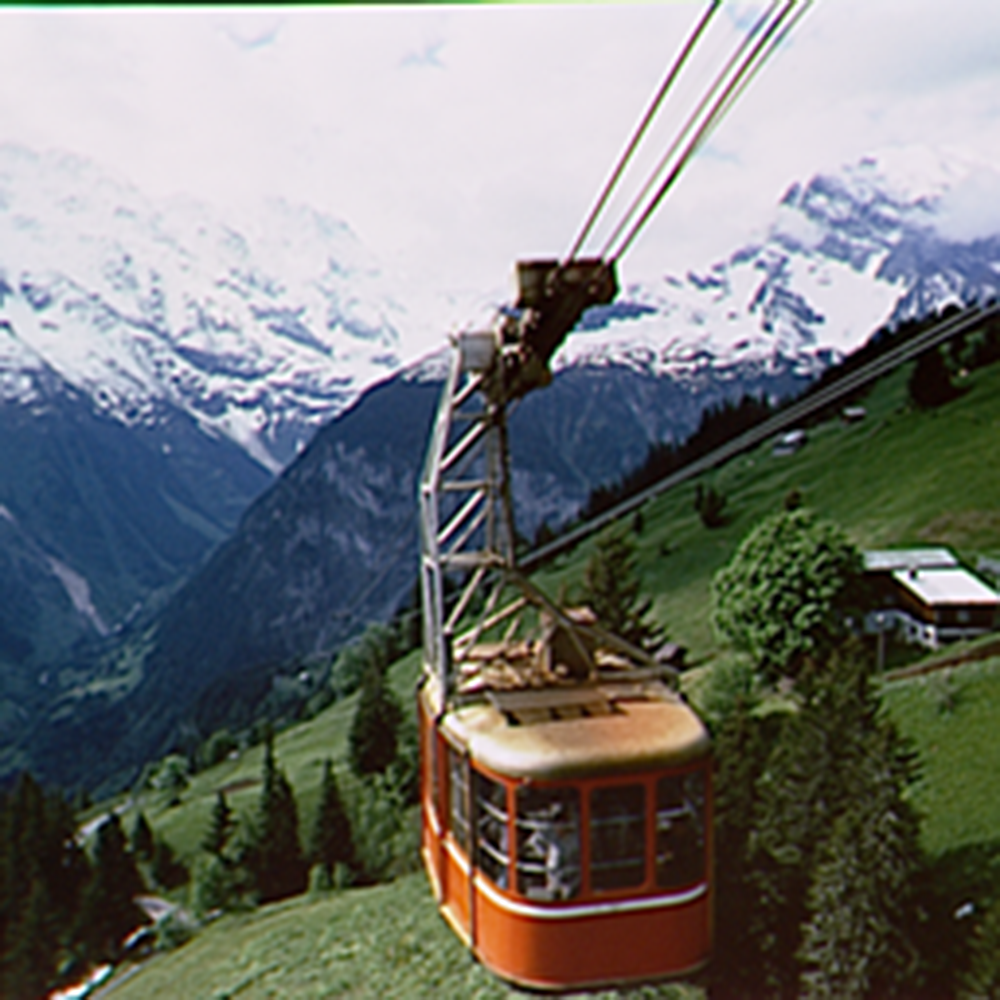

Supplement: S2 Fig — (TIF) [file pone.0184586.s002.tif]

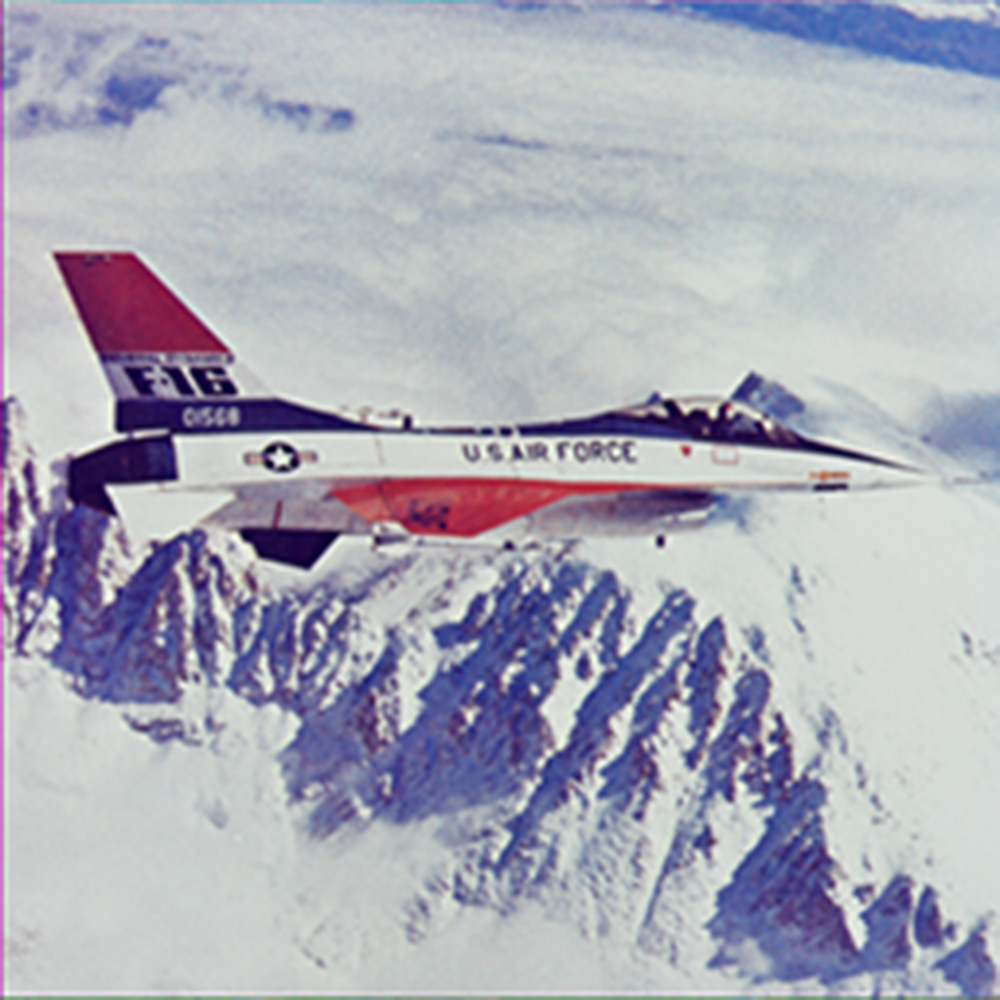

Supplement: S3 Fig — (TIF) [file pone.0184586.s003.tif]

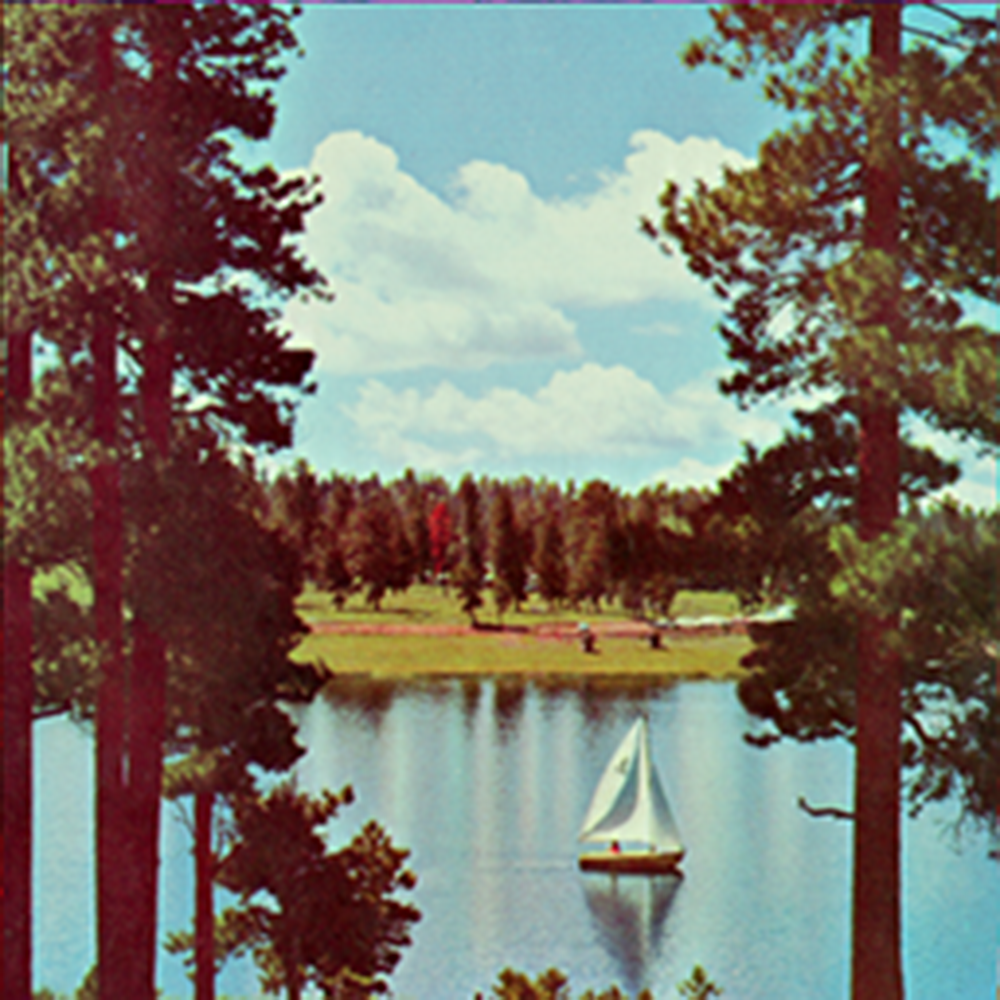

Supplement: S4 Fig — (TIF) [file pone.0184586.s004.tif]

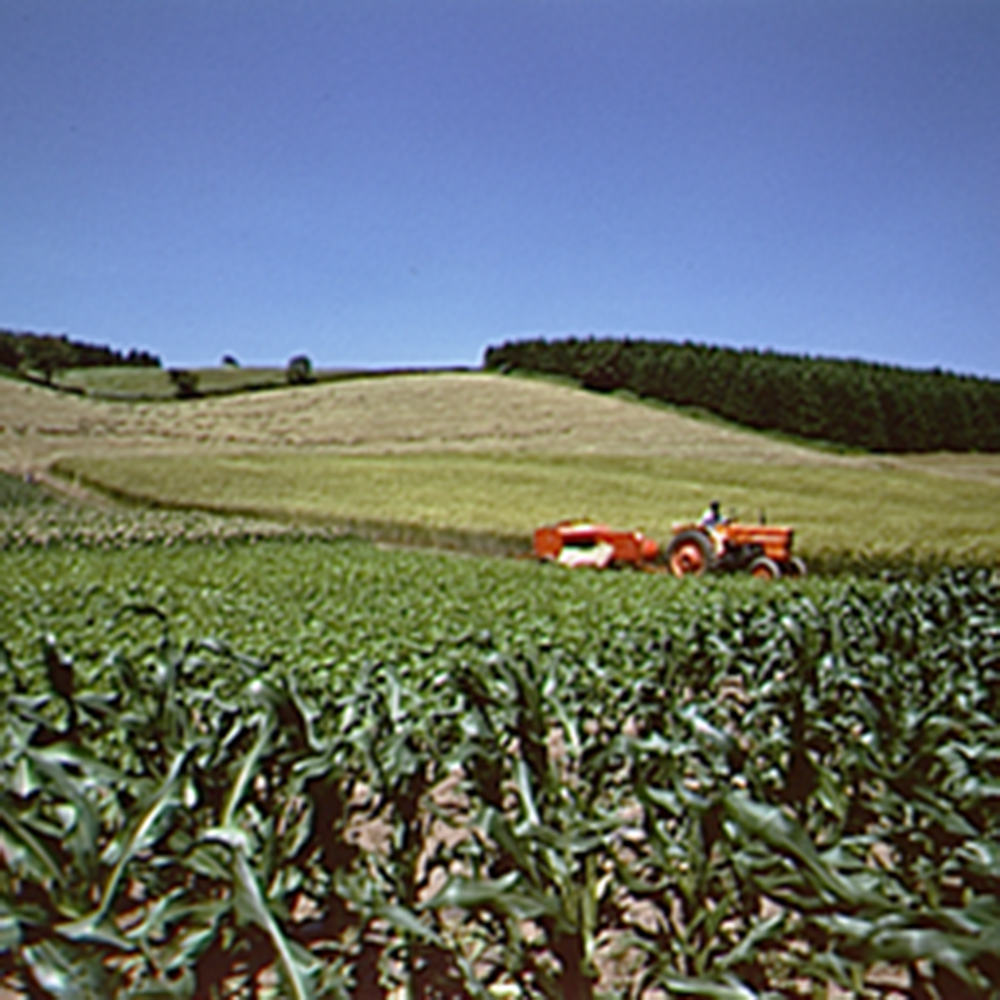

Supplement: S5 Fig — (TIF) [file pone.0184586.s005.tif]

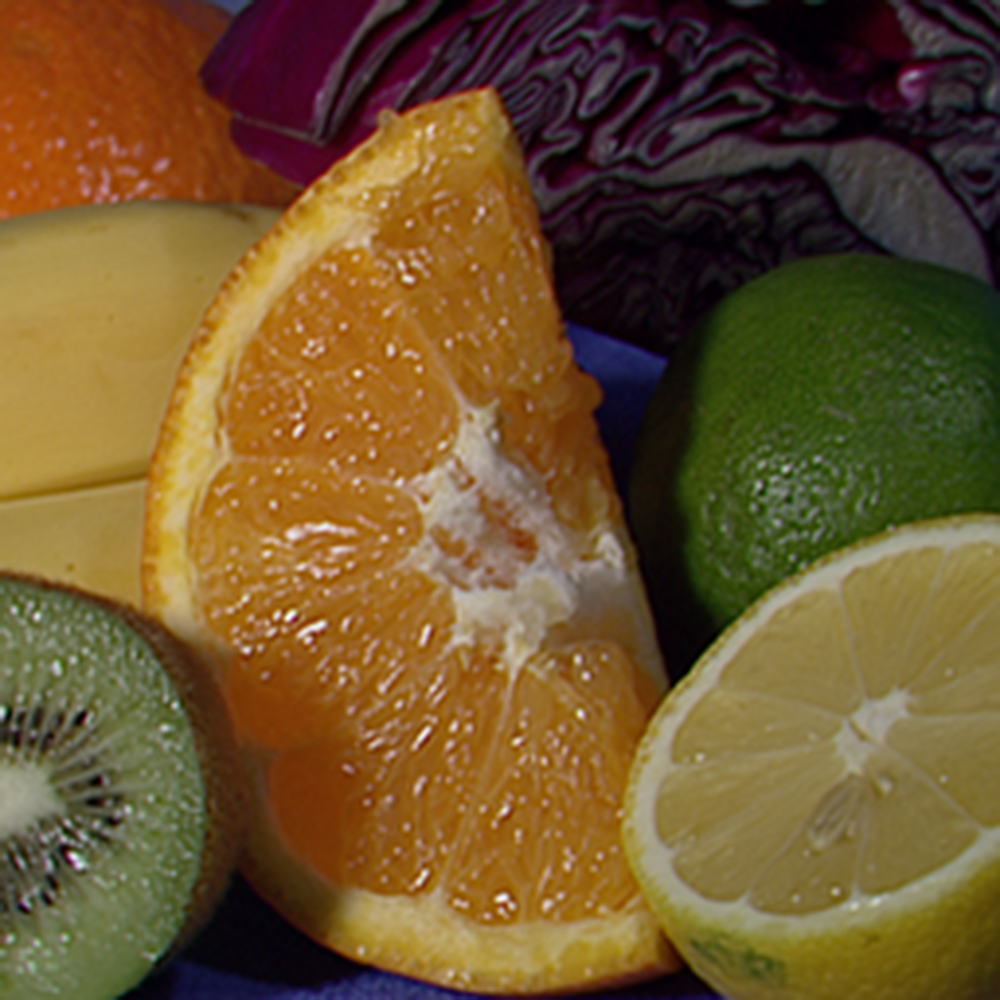

Supplement: S6 Fig — (TIF) [file pone.0184586.s006.tif]

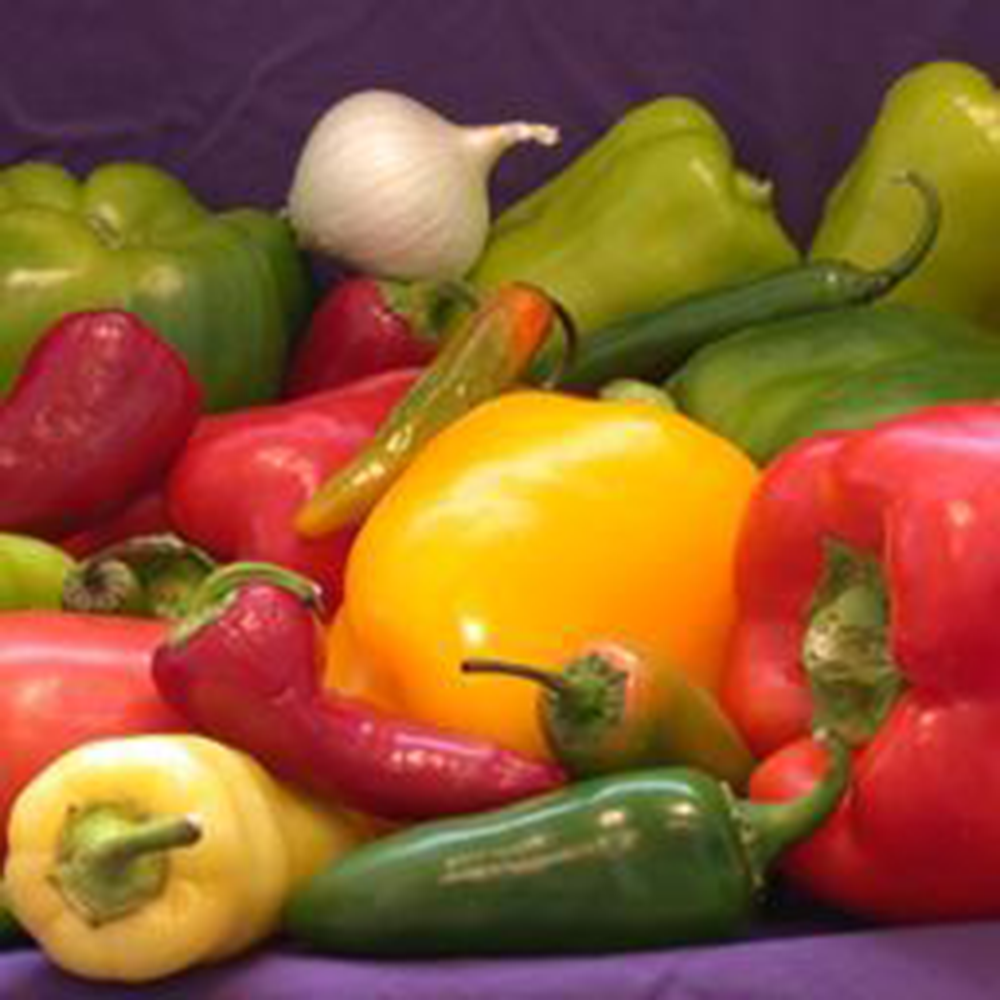

Supplement: S7 Fig — (TIF) [file pone.0184586.s007.tif]

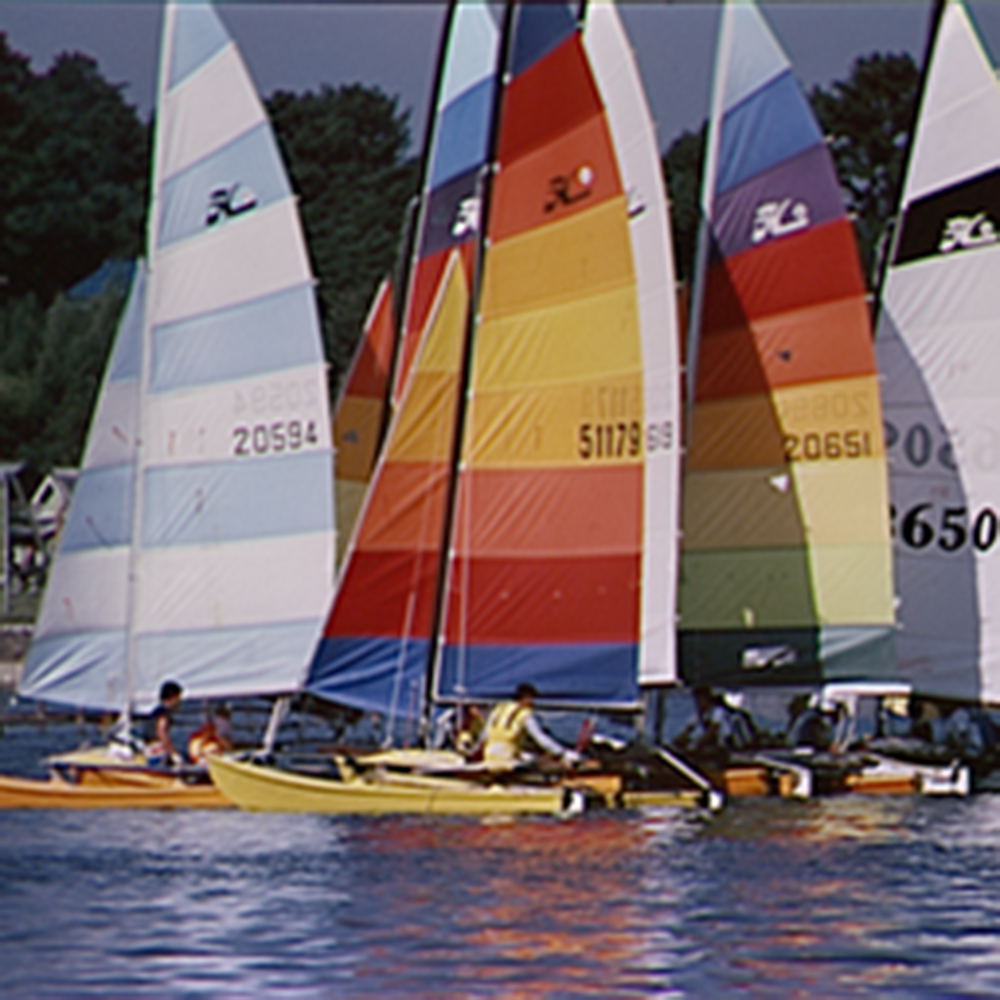

Supplement: S8 Fig — (TIF) [file pone.0184586.s008.tif]
